# Combining phylogenetic and demographic inferences to assess the origin of the genetic diversity in an isolated wolf population

**DOI:** 10.1371/journal.pone.0176560

**Published:** 2017-05-10

**Authors:** Luca Montana, Romolo Caniglia, Marco Galaverni, Elena Fabbri, Atidje Ahmed, Barbora Černá Bolfíková, Sylwia D. Czarnomska, Ana Galov, Raquel Godinho, Maris Hindrikson, Pavel Hulva, Bogumiła Jędrzejewska, Maja Jelenčič, Miroslav Kutal, Urmas Saarma, Tomaž Skrbinšek, Ettore Randi

**Affiliations:** 1 Laboratorio di Genetica, Istituto Superiore per la Protezione e la Ricerca Ambientale (ISPRA), Ozzano dell’Emilia, Bologna, Italy; 2 Institute of Biodiversity and Ecosystem Research, Bulgarian Academy of Sciences, Sofia, Bulgaria; 3 Faculty of Tropical AgriSciences, Czech University of Life Sciences Prague, Prague, Czech Republic; 4 Mammal Research Institute Polish Academy of Sciences, Białowieża, Poland; 5 Department of Biology, Faculty of Science, University of Zagreb, Zagreb, Croatia; 6 CIBIO/InBIO - Centro de Investigação em Biodiversidade e Recursos Genéticos, Campus de Vairão, Universidade do Porto, Vairão, Portugal; 7 Departamento de Biologia, Faculdade de Ciências, Universidade do Porto, Porto, Portugal; 8 Department of Zoology, Institute of Ecology and Earth Sciences, University of Tartu, Tartu, Estonia; 9 Department of Zoology, Charles University in Prague, Prague, Czech Republic; 10 Department of Biology and Ecology, Ostrava University, Ostrava, Czech Republic; 11 Department of Biology, Biotechnical Faculty, University of Ljubljana, Ljubljana, Slovenia; 12 Department of Forest Ecology, Faculty of Forestry and Wood Technology, Mendel University in Brno, Brno, Czech Republic; 13 Friends of the Earth Czech Republic, Olomouc Branch, Olomouc, Czech Republic; 14 Department 18/ Section of Environmental Engineering, Aalborg University, Aalborg, Denmark; Universita degli Studi di Sassari, ITALY

## Abstract

The survival of isolated small populations is threatened by both demographic and genetic factors. Large carnivores declined for centuries in most of Europe due to habitat changes, overhunting of their natural prey and direct persecution. However, the current rewilding trends are driving many carnivore populations to expand again, possibly reverting the erosion of their genetic diversity. In this study we reassessed the extent and origin of the genetic variation of the Italian wolf population, which is expanding after centuries of decline and isolation. We genotyped wolves from Italy and other nine populations at four mtDNA regions (control-region, ATP6, COIII and ND4) and 39 autosomal microsatellites. Results of phylogenetic analyses and assignment procedures confirmed in the Italian wolves a second private mtDNA haplotype, which belongs to a haplogroup distributed mostly in southern Europe. Coalescent analyses showed that the unique mtDNA haplotypes in the Italian wolves likely originated during the late Pleistocene. ABC simulations concordantly showed that the extant wolf populations in Italy and in south-western Europe started to be isolated and declined right after the last glacial maximum. Thus, the standing genetic variation in the Italian wolves principally results from the historical isolation south of the Alps.

## Introduction

Human activities have deeply shaped the structure of landscapes in continental Europe for millennia, often reducing the extension of pristine ecosystems to small isolated fragments [1]. Nowadays habitat fragmentation is a major threat to the survival of natural animal populations [2]. Carnivores and ungulates in particular need wide extensions of suitable habitat and are among the most sensitive species to habitat fragmentation. Ungulates have been intensively hunted for meat while large carnivores were persecuted as pest predators, and both declined for centuries simultaneously with the fragmentation and changes of their natural habitats [3]. However, the ongoing rewilding wave in continental Europe demonstrates that declining population trends can be interrupted and even reversed: endangered top predators and ungulates are now locally abundant, and food chains in forest ecosystems are partially restored [4]. Therefore, it is interesting to understand the consequences of past declines and fragmentation in the perspective of population expansion and forthcoming rejoining.

The genetic consequences of protracted population declines are theoretically well known but not easily predictable in empirical case-studies [5,6]. According to the strength of bottlenecks and time of isolation, genetic diversity (number of alleles, heterozygosity) is progressively lost by random drift. Loss of selectively neutral genetic diversity is correlated to the effective size of the population (*Ne*), which is usually much lower than the observed census population size (*Nc*) [7]. Estimating *Nc* in natural populations is not simple, and estimating *Ne* is even more difficult due to the variability of its several determinants: reproductive success, sex-ratio, departures from random mating and variations of these factors from one generation to the next. Various simulation methods, including the Approximate Bayesian Computation (ABC) [8], have been implemented to evaluate the role of demographic parameters in determining the dynamics of genetic diversity in complex historical scenarios [9]. However, if *Ne* is not too small, the outcomes of genetic drift can be contrasted by balancing or frequency-dependent selection and by hitchhiking on functional gene complexes [10]. Although the fitness consequences of small *Ne* and low standing genetic variation are controversial, the assumption that adaptive potential and evolvability are positively correlated to heterozygosity is a precautionary principle widely accepted in conservation genetics [11].

In this study, we reassessed the amount and origin of genetic variation in a wolf (*Canis lupus*) population that remained isolated and declined in peninsular Italy for centuries [12,13]. Wolves disappeared from the Alps in the early 1900s and were strongly reduced in the peninsular regions until the early 1970s, when less than 100 individuals survived in two isolated sub-populations in remote mountain areas of the central-southern Apennines [14]. Both legal protection (granted to the wolf in the 1970s) and deep socio-ecological changes (industrialization, urbanization, and the abandonment of marginal agricultural lands in mountains and hills) boosted spectacular re-expansions of forests, wild ungulates and consequently wolves [4]. Permanent wolf packs rapidly established along the entire Apennines chain, reaching the western Alps in the early 1990s, then colonizing new areas in lowlands and also in southern Italy. Currently, the Italian wolf population consists of *c*. 1500 (± 300) individuals and at least 320 documented packs [15].

Regardless of the strength of the ongoing wolf expansion trend, the amount of standing genetic variability should have been largely determined by the duration of the last bottleneck, because there has not been enough time for mutations to reconstitute the lost variation [13]. However, the effective isolation period of the Italian wolf population south of the Alps is controversial. Genetic and genomic data suggest that it could have been effectively isolated for 9500–19 000 years (as estimated using microsatellite data, depending on inferred *Ne* values) [13], 3200–5600 years (as estimated from genome-wide SNP data) [16], or 2800–7000 years (from whole genome sequences, depending on the mutation rate applied [17]), much longer than the *c*. 100 years estimated from the observed isolation south of the Alps. Genetic analyses indicate that the autosomal genetic diversity in the Italian wolves is *c*. 30% lower than in other wolf populations in Europe [16]. The mtDNA genetic diversity is limited to a single control-region (CR) haplotype named W14 that is widespread uniquely in the Italian wolves [18], although a recent study suggests that a second rare haplotype named W16 is also naturally present in the population [19]. Monitoring programs and molecular analyses revealed an increasing occurrence of wolf hybridization with free-ranging domestic dogs in sectors of the wolf range in Peninsular Italy [20,21]. The uncertain length of isolation and the occurrence of hybridization make it difficult to understand if the observed genetic diversity is explained by the last bottleneck or by admixtures.

In this study we genotyped wolves sampled from the Italian and nine other populations in Europe at four mtDNA regions (the control-region CR, and three coding genes: ATP6, COIII and ND4) and 39 autosomal microsatellites [20]. Aiming to identify possibly-admixed genotypes and clarify the origin of the observed genetic diversity, we included also 69 village dogs and 74 known wolf *x* dog hybrids sampled from areas within the wolf distribution range in Italy and Estonia. Specifically, we aimed to: 1) confirm the attribution of the rare mtDNA CR haplotype W16 to the Italian wolf population [19], increasing the sample size of wolves and hybrid canids from different European population, and 2) test if the standing genetic variation in the Italian wolves is mainly determined by a historical isolation south of the Alps dating back to the end of the last Pleistocene glaciation, or to the most recent anthropogenic bottleneck about one century ago. In order to validate the most likely hypotheses, we performed Bayesian assignment analyses to investigate the partition of autosomal genetic diversity among wolf populations, then we applied Bayesian phylogenetic procedures to estimate the divergence times among wolf and dog mtDNA sequences and ran ABC simulations to reconstruct past demographic scenarios and infer splitting dates of extant wolf populations in Europe.

## Materials and methods

### Ethics statement

No animals were sacrificed for the only purposes of this study. In Italy, all samples of found-dead wolves were collected by specialised technician personnel on behalf of the Italian Ministry of Environment (MATTM) and Italian Institute for Environmental Protection and Research (ISPRA). Wolf stool samples from Czech Republic and Slovakia were collected by Friends of the Earth organisation (FoE CZ), which is monitoring the wolf population in the Carpathian Mountains. In Slovakia, FoE CZ has permission to collect non-invasive samples of wolves, issued by Regional Office Trenčín, Department of Environment, No. OU-TN-OSZP1-2014/49/3475. Carpathian wolf tissue samples were legally culled during the open hunting season in Slovakia within a quota set by the local authorities, in conformity with regulation No 344/2009 Coll. The wolves were shot during individual patrols or collective hunts. The use of poisoned bait or leg-hold traps is strictly forbidden according to hunting law. All hunters had permission for hunting, and we confirmed that the culls were reported before quota fulfilment. Croatian and Slovenian samples were obtained either from animals killed in traffic accidents or culled during regular hunting management according to quotas defined by the Croatian Commission for monitoring large carnivore populations and approved by the Croatian Ministry for Environmental and Nature Protection. Iberian wolf samples from Portugal were from found-dead individuals collected by specialised technician personnel on behalf of the Portuguese Institute for Nature Conservation (ICNF). Wolf samples from Spain were obtained from animals that were road-killed or culled during regular hunting management according to quotas defined by Principado de Asturias authorities. Samples from Estonia, Latvia, Finland, Poland, Greece, and Bulgaria were also collected from animals found dead or legally harvested by hunters for purposes other than this project. No ethics permit was required since the sample collection involved dead animals. All samples were collected by specialised technician personnel. No ethic permit is also required to collect stool samples in these countries. Dog blood samples were obtained by veterinaries with the assistance of the owners and all the possible efforts to minimize animal suffering. The owners of the dogs gave permission for their animals to be used in this study. Salivary samples were obtained through buccal swabs by specialised technicians.

### Sampling

We genotyped 190 wild-living wolves, 69 village dogs and 74 known wolf *x* dog hybrids from Italy and several other countries in Europe (Table 1). Moreover, we analysed other five unrelated wild-living wolves, sampled in different areas of the central-northern Apennines, that had the rare mtDNA haplotype W16 [19,20] (Table 1). Wolf samples were collected from 1990 to 2015 in Italy (sample size *n* = 34), Spain and Portugal (Iberian Peninsula; *n* = 20), Slovenia (20), Croatia (20), Greece (15), Bulgaria (17), Czech Republic and Slovakia (20), Poland (16), Estonia (10), Latvia (10), and Finland (9). The 69 village dogs were sampled in Italy from northern and central Apennine areas, had size and shapes similar to shepherd dogs, did not belong to certified breeds, and were selected independently of genotypic information. The known wolf *x* dog hybrids were sampled in Italy (68) and Estonia (6), and were previously identified by genetic or morphological analyses [20,22]. All wolves had the typical wolf coat colour pattern and did not show any apparent morphological sign of hybridization. We stored tissue and blood samples in 10 volumes of 95% ethanol or five volumes of a Tris/SDS buffer [23] at -20°C, respectively. We extracted DNA samples using the QIAGEN DNeasy tissue extraction kit (Qiagen Inc, Hilden, Germany) in a robotic liquid handling system MULTIPROBE II^EX^ (Perkin-Elmer, Weiterstadt, Germany). All the DNA samples used in previous studies were reanalyzed. Negative extraction controls (no DNA in the test tubes) were used to check for laboratory contaminations.

**Table 1:** Country of origin and size of the wolf, dog and wolf *x* dog hybrid samples analyzed in this study.

Taxon	Country	Genetic cluster^a^	Acronym	mtDNA^b^	STR^c^	Total
**Wolves**	Italy^d^	Italian wolves	WIT	39	39	39
	Portugal, Spain	Iberian wolves	WIB	20	20	20
	Slovenia	Dinaric wolves	WDIN	20	20	20
	Croatia^e^	Dinaric wolves	WDIN	---	20	20
	Greece	Balkanic wolves	WBALK	15	10	15
	Bulgaria	Balkanic wolves	WBALK	17	17	17
	Czech & Slovakia^e^	Carpathian wolves	WCARP	---	20	20
	Poland^f^	---	---	16	---	16
	Estonia	Baltic wolves	WBALT	10	10	10
	Latvia	Baltic wolves	WBALT	10	10	10
	Finland	Baltic wolves	WBALT	9	9	9
**Dogs**	Italy	Italian dogs	DIT	8	69	69
**Hybrids**	Estonia	Hybrids	HY	6	6	6
	Italy	Hybrids	HY	40	68	68
**Total**				210	318	339

^a^ Genetic clusters and their acronyms, as defined by Bayesian cluster analyses (see: Results);

^b^ mtDNA = samples sequenced at the mtDNA CR, ATP6, COIII and ND4 regions;

^c^ STR = samples genotyped at 39 autosomal microsatellite (STR);

^d^ The five wild-living wolves, sampled in Italy, showed a rare mtDNA haplotype, named W16 [18] and recently attributed to the Italian wolf population [19];

^e^ Wolves from Croatia, Czech Republic and Slovakia were only used in Bayesian cluster analyses and ABC simulations;

^f^ Wolves from Poland were not genotyped at the STR loci due to their low DNA quality, and were not assigned to any genetic cluster.

### Microsatellite genotyping

We selected a panel of 39 canine autosomal microsatellites (seven tetranucleotides and 32 dinucleotides) that were used in some of the most recent studies on wolf population genetics and hybridization in Europe ([24] and references therein). These microsatellites mapped on 26 different chromosomes (S1 Table) and were not in linkage disequilibrium in the studied populations. The panel includes 15 markers from the Finnzymes Canine Genotypes^™^ Panel 1.1 multiplex kit (Finnzymes, Thermo Fisher Scientific, Vantaa, Finland). One of them, the *Amelogenin*, was used to sex the individuals. The microsatellites were amplified in eight PCR multiplexes using the Qiagen Multiplex PCR Kit (Qiagen, GmbH-Hilden, Germany). Negative (no DNA) and positive (samples with known genotypes) PCR controls were used to check for laboratory contaminations. To confirm allele calls, all samples were independently analysed twice to check for the occurrence of allelic dropout and false alleles, which were never observed. The amplicons were analysed in an ABI 3130XL automated sequencer (Applied Biosystems; Foster City, California, USA) and allele sizes were estimated using the software Genemapper 4.0. Details on the selected markers, primers and PCR profiles are available in the S1A Appendix.

### Microsatellite variability, genotype clustering and assignment testing

Observed and effective allele numbers (*Ao* and *Ae*), observed and expected heterozygosity (*Ho* and *He*), *F*-statistics [25], and tests for departures from Hardy-Weinberg (HWE) and linkage equilibria (LE) were computed in GenAlEx 6.01 [26] and Genetix 4.05 [27]. The individual multilocus genotypes were clustered and assigned to their most likely population of origin using: 1) a principal coordinate analysis (PCoA in GenAlEx); 2) a discriminant principal component analysis (DAPC in Adegenet) [28]; and 3) a Bayesian clustering model (minimizing departures from HWE and LE in the genetic clusters) implemented in Structure 2.3.4 [29,30]. We used Structure to infer the optimal genetic partition of the sampled groups, assuming *K* from 1 to 15 with four independent runs for each *K* with 500 000 Monte Carlo Markov Chains (MCMC) steps and discarding the first 50 000 steps as burn-in, using the *admixture* and *independent allele frequency* models, and no prior information (option *usepopinfo* not activated). We used Clumpak (http://clumpak.tau.ac.it) to identify the highest rate of increase in the posterior probability Ln*P*(D) of the clusters between each consecutive *K* [31] and to aggregate the individual membership probability (*q*i) from the four MCMC replicates [32].

### Mitochondrial DNA sequencing

We amplified by Polymerase Chain Reaction (PCR) a fragment of the left peripheral and central domains of the mtDNA control-region (CR) using primers WDLOOPL and H519 [33], and fragments of the mtDNA ATP6, COIII and ND4 genes using primer pairs described in [34] (primers For8049-Rev8501; For8255-Rev8891; For10104-Rev10647; For11093-Rev11741). All the amplifications were performed in 10 μL total reactions containing 20–40 ng/μL DNA, 1X PCR buffer with 2.5 mM Mg^2+^, 0.3 μM of primer mix (forward and reverse) and 0.25 units of Taq Polymerase (5 PRIME Inc., Gaithersburg, USA). Amplifications were performed with an initial DNA denaturation step at 94°C for 2 minutes, followed by 45 cycles of denaturation at 94°C for 15 seconds, annealing at 55°C for 15 seconds, extension at 72°C for 30 seconds, and final extension at 72°C for 10 minutes. Amplicons were purified using ExoSAP-IT (Affimetrix, Inc., Cleveland, Ohio, USA) and sequenced in both directions in an ABI automated DNA sequencer 3130XL (Applied Biosystems). Sequences were visually corrected in SeqScape 2.5 and aligned in Geneious 7.1 (Biomatters Ltd., Auckland, New Zealand). Geneious was also used to fix alignment ambiguities, mainly caused by indels in the mtDNA CR. The four mtDNA regions were concatenated in a multi-fragment alignment of 2164 bp. Taking into account the presence of indels, identical haplotypes were collapsed using DnaSP 5.10.01 [35], that was also used to estimate haplotype (*H*) and nucleotide (π) diversity [36] for each of the four regions and for the concatenated sequences.

### Phylogenetic analyses

In addition to the new 210 mtDNA sequences (S2 Table), we downloaded from GenBank the homologous sequences of 18 extant wolves, four ancient canids and 322 dogs (S3 Table). We then aligned these sequences to construct Neighbor-Joining (NJ) [37], Maximum Likelihood (ML with heuristic search) [38] and Bayesian (BT) [39] phylogenetic trees that were rooted using as an outgroup a coyote sequence (*Canis latrans*, GenBank access number DQ480509). NJ and ML phylogenetic analyses were done in Paup* 4.0 beta [40]; the BT was computed in MrBayes 3.2 [41]. For the NJ and ML analyses, we selected the best evolutionary model using the *modeltest* option [42] and the Akaike Information Criterion [43]. We obtained internode supports by 1000 bootstrap replicates [44] in NJ trees, and by 100 bootstrap replicates in ML trees, using the *faststep* search in Paup. We identified the best-fit evolutionary model for Bayesian analyses using PartitionFinder [45]. MrBayes 3.2 was run for 2x10^6^ generations, with a sampling frequency of 100 generations, and with one cold and three heated MCMC (temperature = 0.45; first 10% of the trees excluded to ensure convergence) [46]. To check for convergence of parameter for Bayesian analyses, we used Tracer 1.6 (http://tree.bio.ed.ac.uk/software/tracer). We estimated in Geneious the frequency of invariable (*I*) sites, the parameters of the gamma (*γ*) distributions and the transition-transversion (*Ti/Tv*) ratios for the NJ, ML, and BT analyses.

### Estimates of mtDNA haplotype divergence times

We used Beast 2.4.2 [47] to estimate the coalescent time to the most recent common ancestor (TMRCA) of the main mtDNA clades identified in the phylogenetic trees of the concatenated sequences, including all the wolf and dog haplotypes. We applied a strict molecular clock with the *coalescent extended bayesian skyline* model [48], and using as priors the ages of the four ancient canid sequences (S3 Table; S1B Appendix). Model parameters and trees were sampled every 10 000 over a total of 100 000 000 iterations in two independent MCMC chains. The first 10% iterations were discarded as burn-in. We used Tracer to check for MCMC convergence. When the two independent runs converged on the posterior distributions and reached stationarity, we combined the sampled trees into a single tree file with LogCombiner 2.4.2 (burn-in = 10%). With TreeAnnotator 2.4.2 we summarized information from a sample of trees into a single final tree visualized in FigTree 1.4.2 (http://tree.bio.ed.ac.uk/software/figtree/). LogCombiner and TreeAnnotator are part of the Beast package.

### ABC simulations

We used microsatellite data to run Approximate Bayesian Computation simulations (ABC) [49] implemented in the software Diyabc 2.1.0 [50] to model plausible demographic scenarios and estimate divergence times (in generations) among wolf populations sampled from European countries and corresponding to the clusters identified by Structure.

We selected three wolf population samples for modelling full ABC simulation scenarios: WIT (pop1), WIBP (pop2) and WDIN (pop3), excluding any sample with possible traces of dog admixture (S2 Table). Samples from WBALK, WCARP and WBALT were not used in the simulations because these populations are still in connection one another and also with unsampled wolf populations in eastern Europe [51,52].

According to Pilot et al. [16] and Fan et al. [53], southern European populations diverged very closely in time, and their effective sizes steadily decreased in the last tens of thousands of years. Therefore, we tested four demographic scenarios (S5 Fig), assuming that the three populations split simultaneously (scenarios 1 and 2) or sequentially (scenarios 3 and 4) and that the three populations passed through a bottleneck (scenarios 2 and 4) or not (scenarios 1 and 3).

We ran 6 x 10^6^ simulations for each scenario using uniform prior distributions of the effective population size and time parameters with default mutation settings. We selected the following summary statistics for all the microsatellites: a) one sample: mean number of alleles, mean genetic diversity, mean size variance; b) two samples: mean number of alleles, mean genetic diversity, *Fst*, shared allele distance (S4 Table).

Scenarios were compared by estimating posterior probabilities with the logistic regression method in DIYABC using 1% of the simulated datasets. For the best models, posterior distributions of the parameters were estimated with a logit-transformed linear regression on the 1% simulated datasets closest to the observed data. Scenario confidence was evaluated by comparing observed and simulated summary statistics. Finally, the goodness-of-fit of the posterior parameters for the best performing scenarios was tested via the model checking option with default settings, and significance was assessed after Bonferroni correction for multiple testing [54].

## Results

### Microsatellite variability and cluster analysis

**Fig 1:**
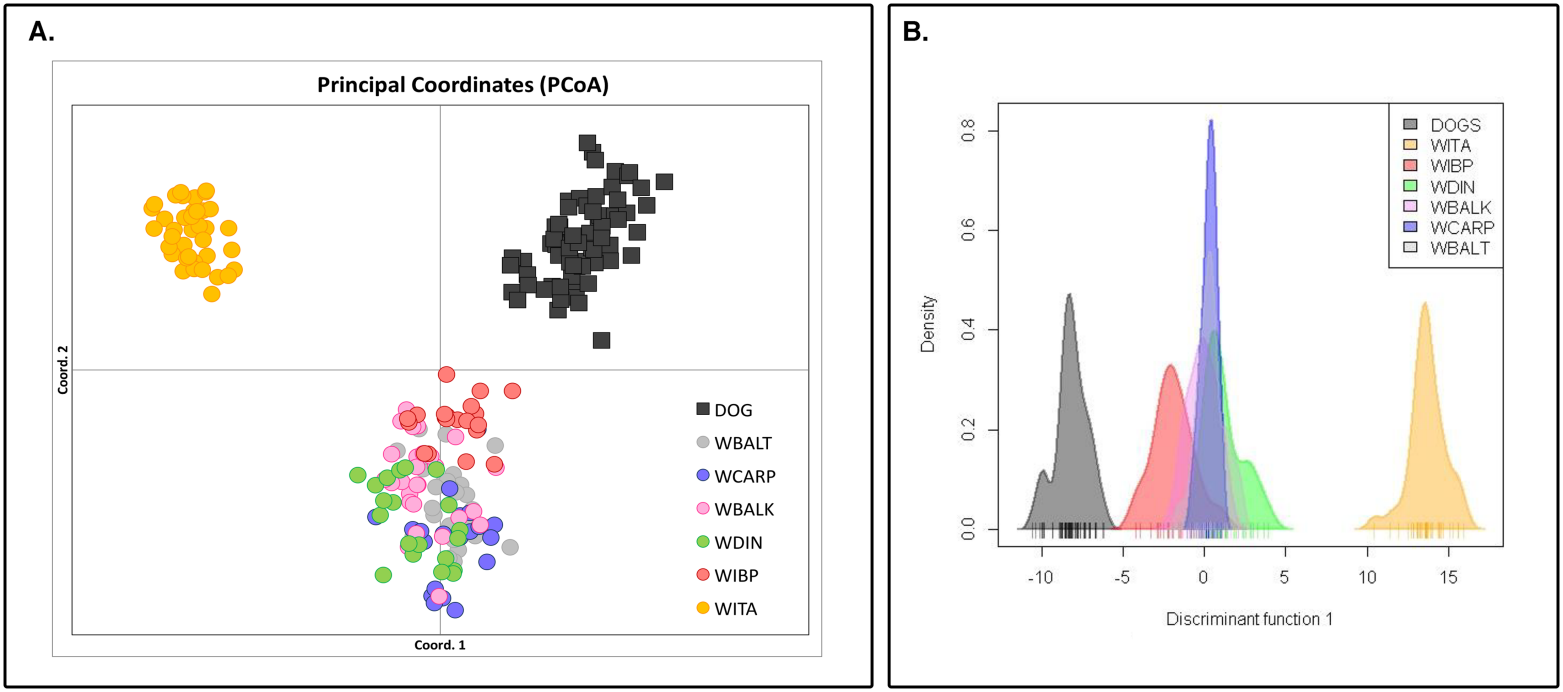
Principal coordinate analysis of multilocus microsatellite wolf and dog genotype. (A) First two components of a PCoA computed in GenAlEx [26] of the 39 multilocus microsatellite wolf and dog genotypes. (B) Multilocus microsatellites wolf and dog genotypes projected on the first function of a discriminant PC analysis (DAPC computed in Adegenet [28]). Identification of wolf samples: WBALT = Baltic countries; WCARP: Carpathians; WBALK = Balkans; WDIN = Dinarics; WIBP = Iberian Peninsula; WITA = Italy.

**Fig 2:**
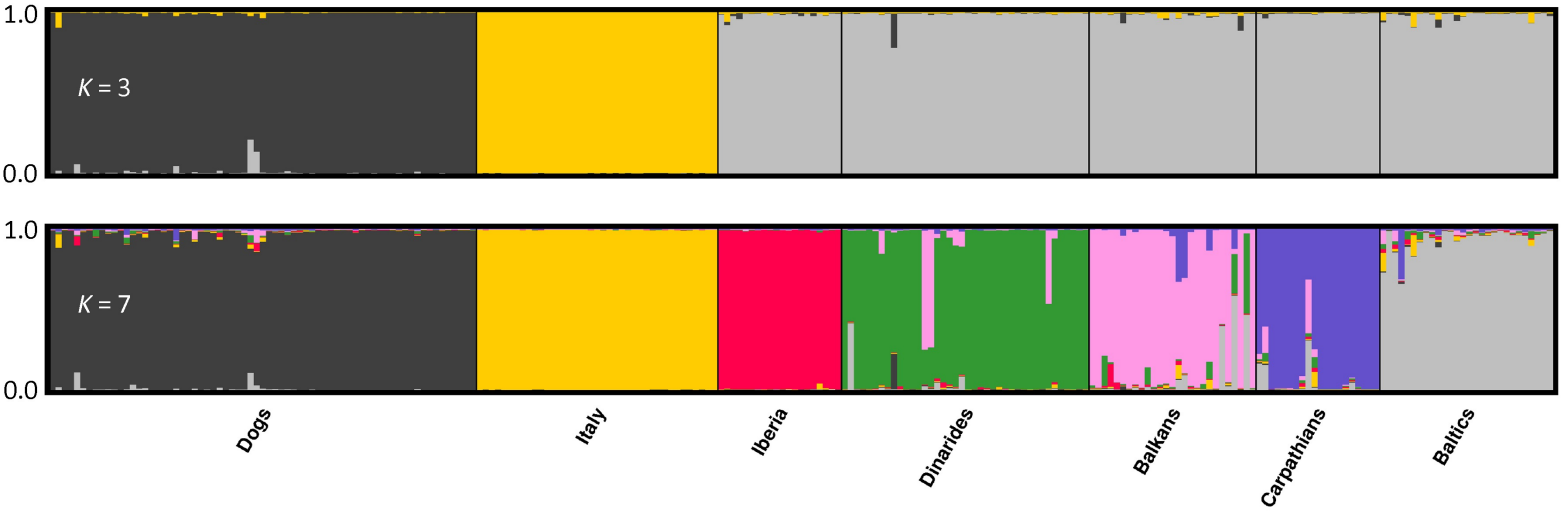
Bayesian clustering of dog and wolf samples from different countries genotyped with 39 autosomal microsatellite loci obtained by Structure [29,30] assuming *K* = 3 and *K* = 7. At *K* = 3 the three clusters are composed by dogs, Italian wolves and by all the other European wolves banded together, while at *K* = 7 wolves are split into six different geographical population clusters.

All the 39 microsatellites were polymorphic in the sampled groups, confirming previously published results [20]. The PCoA plotting of the multilocus genotypes showed that the Italian wolves and dogs are sharply distinct from one another and from all the other wolf populations (Fig 1A). The DAPC plot confirmed this pattern of population clustering (not shown). The first discriminant function in DAPC further showed that wolves from the Iberian Peninsula clustered in a separate sector from the other regions in Europe (Fig 1B). Results of the multivariate analyses are supported by Structure results, where the best clustering were obtained at *K* = 3 and at *K* = 7, wherein the optimal subdivision for the European wolf populations was observed (Fig 2; S1 Fig). Dogs and wolves cluster separately at *K* = 2, and the Italian wolves were the first to cluster separately from the other wolves at *K* = 3, followed by the Iberian wolves at *K* = 4, then by wolves from Slovenia and Croatia at *K* = 5, from Greece and Bulgaria at *K* = 6, from Carpathian region, Estonia, Latvia and Finland at *K* = 7 (S2 Fig). We assume that these seven clusters represent the main genetic subdivision among the sampled populations, and hereafter we will refer to the following wolf populations: Italian Peninsula (WIT), Iberian Peninsula (Spain and Portugal; WIB), Dinaric regions (Slovenia and Croatia; WDIN), Balkanic regions (Greece and Bulgaria; WBALK), Carpathians (Czech Republic; WCARP), and Baltic Countries (Estonia and Latvia plus Finland; WBALT) (Table 1). WIT and WIB did not show signatures of admixed ancestry, which, however, was apparent in some individuals sampled in other clusters (Fig 2). The five wolves carrying the W16 CR haplotype were totally assigned to the WIT cluster with *q*_*wit*_ > 0.99 and 90% CI = 0.95–1.00 (samples W943, W1223, H1122, W1816 and W1906 listed at the end of S2 Table) and were confirmed to belong to the WIT population.

The numbers of observed and effective microsatellite alleles were the lowest in WIT (*Ao* = 3.9; *Ae* = 2.3) and the highest in WBALT (*Ao* = 6.7; *Ae* = 4.1). These two populations also showed the lowest (WIT: *Ho* = 0.44; *He* = 0.50) and the highest (WBALT: *Ho* = 0.68; *He* = 0.73) values of heterozygosity. Values of *Ho* were slightly smaller than expected in all the population clusters except WCARP that showed identical *Ho* and *He* values. Thus, the *F*-values within clusters were positive, indicating departures from HWE (Table 2).

**Table 2:** Estimated genetic variability in six wolf clusters identified by Bayesian analyses.

	Microsatellites		mtDNA						
					CR	ND4	COIII	ATP6	MF
**WIT**	n	39	n	39					
	Ao/Ae	3.9/2.3	N		2	1	1	1	2
	Ho/He	0.44/0.50	H		0.229	0.000	0.000	0.000	
	F	0.117	π		0.00046	0.00000	0.00000	0.00000	
**WIB**	n	20	n	20					
	Ao/Ae	4.5/3.1	N		4	3	2	1	5
	Ho/He	0.52/0.61	H		0.711	0.416	0.100	0.000	
	F	0.127	π		0.00299	0.00052	0.00043	0.00000	
**WDIN**	n	40	n	20					
	Ao/Ae	6.1/3.6	N		2	2	2	2	3
	Ho/He	0.62/0.69	H		0.337	0.337	0.337	0.337	
	F	0.117	π		0.00747	0.00119	0.00583	0.00286	
**WBALK**	n	27	n	32					
	Ao/Ae	6.6/3.9	N		7	6	3	3	10
	Ho/He	0.66/0.71	H		0.843	0.760	0.589	0.679	
	F	0.085	π		0.01408	0.00208	0.00866	0.00448	
**WCARP**	n	20	n	n.a.					
	Ao/Ae	4.7/3.1	N						
	Ho/He	0.64/0.64	H						
	F	0.000	π						
**WBALT**	n	28	n	28					
	Ao/Ae	6.7/4.1	N		5	5	3	2	6
	Ho/He	0.68/0.73	H		0.605	0.510	0.446	0.353	
	F	0.073	π		0.00947	0.00304	0.00763	0.00699	

Cluster composition and acronyms are described in Table 1. Microsatellites = 39 autosomal microsatellites; n = genotyped samples; Ao/Ae = average observed/effective number of alleles; Ho/He = average observed/expected heterozygosity; F = inbreeding coefficient. mtDNA = sequences at CR, ND4, COIII and ATP6 mtDNA regions; n = sequenced samples; N = haplotype numbers; H = haplotype diversity; π = nucleotide diversity. MF = number of concatenated multi-fragment haplotypes detected in the six wolf clusters.

### Phylogenetic analyses

We sequenced 498 bp of the mtDNA CR (GeneBank accession number KY549989-KY550013), 588 bp of the ATP6 (GeneBank accession no. KY549946-KY549953), 231 bp of the COIII (GeneBank accession no. KY549954-KY54960) and 847 bp of the ND4 (GeneBank accession no. KY549961-KY549974 for the first 414 bp and no. KY549975-KY549988 for the last 433 bp) mitochondrial genes in 210 canid samples (2164 bp in total; S2 Table). The Italian wolves (WIT) showed the lowest number of haplotypes (*N*), and the lowest haplotype and nucleotide diversity (*H* and π) at each of the four mtDNA regions (Table 2). Nucleotide diversity was lower than 1.4% in all the sampled populations, suggesting recent origins of their mtDNA diversity. The concatenated mtDNA sequences yielded two distinct haplotypes in the Italian wolves, five in WIB, three in WDIN, 10 in WBALK and six in WBALT (Table 2). The two Italian wolf haplotypes, WH14 and WH19, differed by a single nucleotide substitution at position 15 629 of the complete *Canis lupus* mitochondrial genome (GenBank access no AB499825). Haplotype WH14 includes the shorter mtDNA CR haplotype W14 repeatedly described in the Italian wolf population [13,18,20,52]. The concatenated haplotype WH19 includes the shorter mtDNA CR haplotype W16 already described by Boggiano et al. [55] and Randi et al. [20] (S2 and S5 Tables) in five of our analyzed samples. We did not detect WH19 in any other individual (S6 Table). All the 40 sequenced wolf *x* dog hybrids sampled in Italy showed the same wolf haplotype WH14, suggesting a preferential wolf maternal-biased hybridization [22].

The BT, NJ and ML phylogenetic trees of the concatenated mtDNA sequences showed very similar topologies, although node supports and relationships among the less divergent haplotypes differed (S3A, S3B and S4 Figs). The two closely related Italian wolf haplotypes WH14 and WH19 belong to the same basal clade named A1 in the phylogenetic trees. Clade A1 is strongly supported in the NJ (bootstrap = 84.7), ML (bootstrap = 70) and BT (posterior probability = 1.0) trees, and includes other five haplotypes (named WH15, WH17, WH18, WH20, and WH21) that were sampled in wolves from Slovenia, Greece, Bulgaria and Poland. All these haplotype share the same ATP6 (A3) and COIII (C3) haplotypes, but WH14, WH17 and WH19 are the only haplotypes that carry the ND4 haplotype N5, whereas the other four haplotypes carry the haplotype N4 (S5 Table). None of these haplotypes were found in any other extant wolf population analysed so far. Clade A1 is the sister clade of the strongly supported clade A2 that includes five haplotypes found only in ancient dog breeds (Swedish and Norwegian elkhounds; S3 Table) and the haplotype S14.5k that was identified in an ancient wolf sample from Switzerland dated at *c*. 14 500 years ago [56]. Clade A is a sister clade to all the other modern wolf and dog clades in the BT, suggesting that it includes ancient mtDNAs currently distributed mainly in southern European wolf populations. Clades A1-A2 are closely related to, but not nested within, haplogroup A2 as defined by Pilot et al. [57]. With a few exceptions, the other clades are composed exclusively of dog or wolf haplotypes [58,59].

### Bayesian estimates of the mtDNA TMRCA

**Fig 3:**
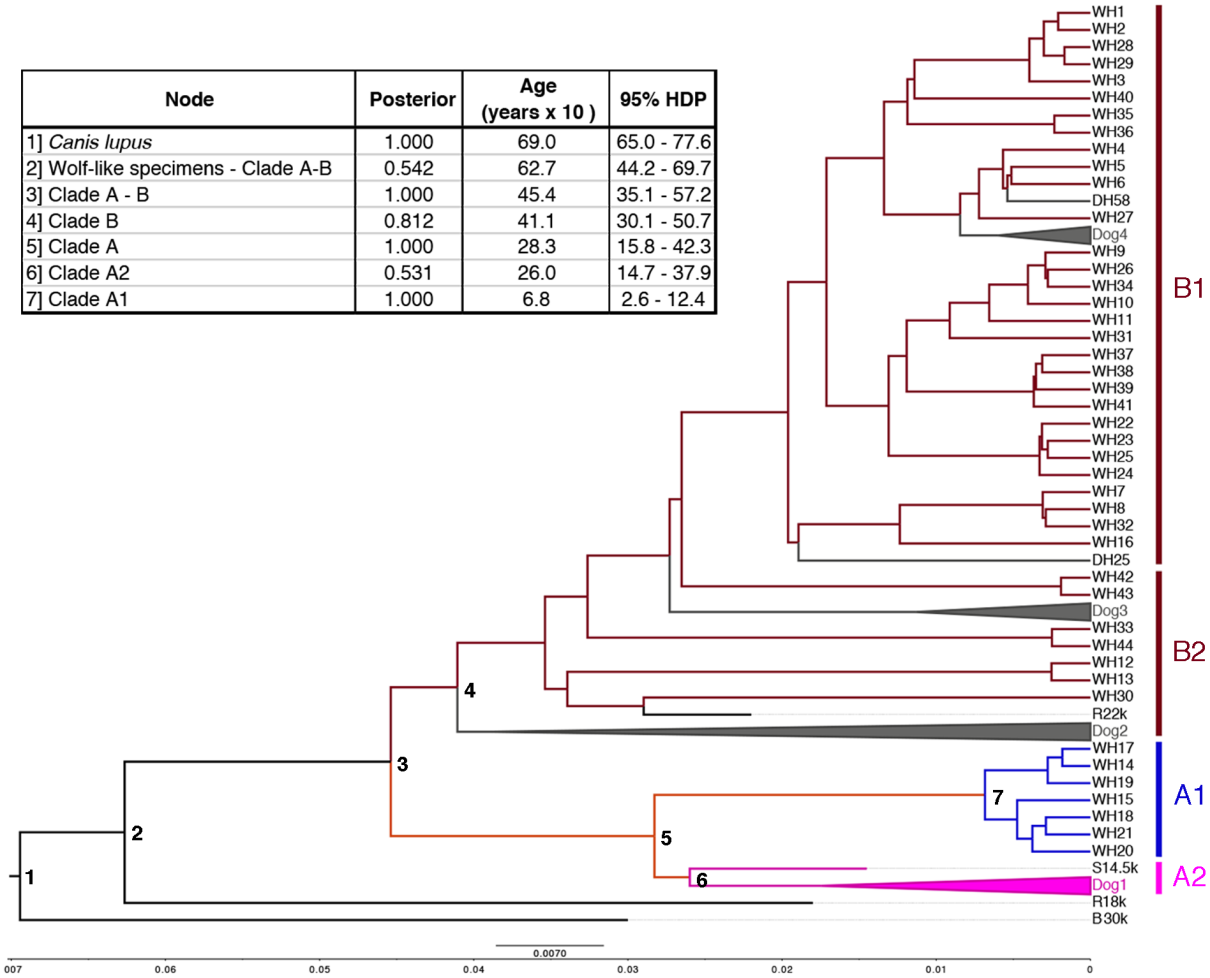
Bayesian mtDNA phylogenetic tree (computed in Beast) [47] with a table indicating the bootstrap support and the estimated TMRCA (and their 95% HDP) of the main internodes. The four main clades A1, A2, B1 and B2 are indicated. All dog haplotypes that form a monophylum were collapsed (see S3 and S5 Tables for the details on the haplotypes and clade composition).

The Bayesian tree generated by Beast (Fig 3) was similar to the other trees. All the posterior probabilities of the main internodes were > 0.81, except for node 2 (P = 0.54) and node 6 (P = 0.53). Consistently with Thalmann et al. [56] the ancient wolf-like specimens from Belgium (B30k) and Russia (R18k) were the most basal haplotypes in the tree (node 1 and 2. The extant wolf haplotypes split into the two clades A and B 45 400 years ago (node 3). Haplotypes within clade B coalesced 41 100 years ago (node 4), whereas clade A1 and A2 coalesced 28 300 years ago (node 5). Clade A2 includes the historical sequence S14.5k [56] and the dog haplotypes Dog1, that diverged 26 000 years ago, whereas the haplotypes included in clade A1 coalesced 6800 years ago (node 7).

### Results of the ABC simulations

ABC simulations provided the best support for scenario 2 (simultaneous population splitting with bottlenecks) that clearly better performed than the other three (S6 Fig).

**Fig 4:**
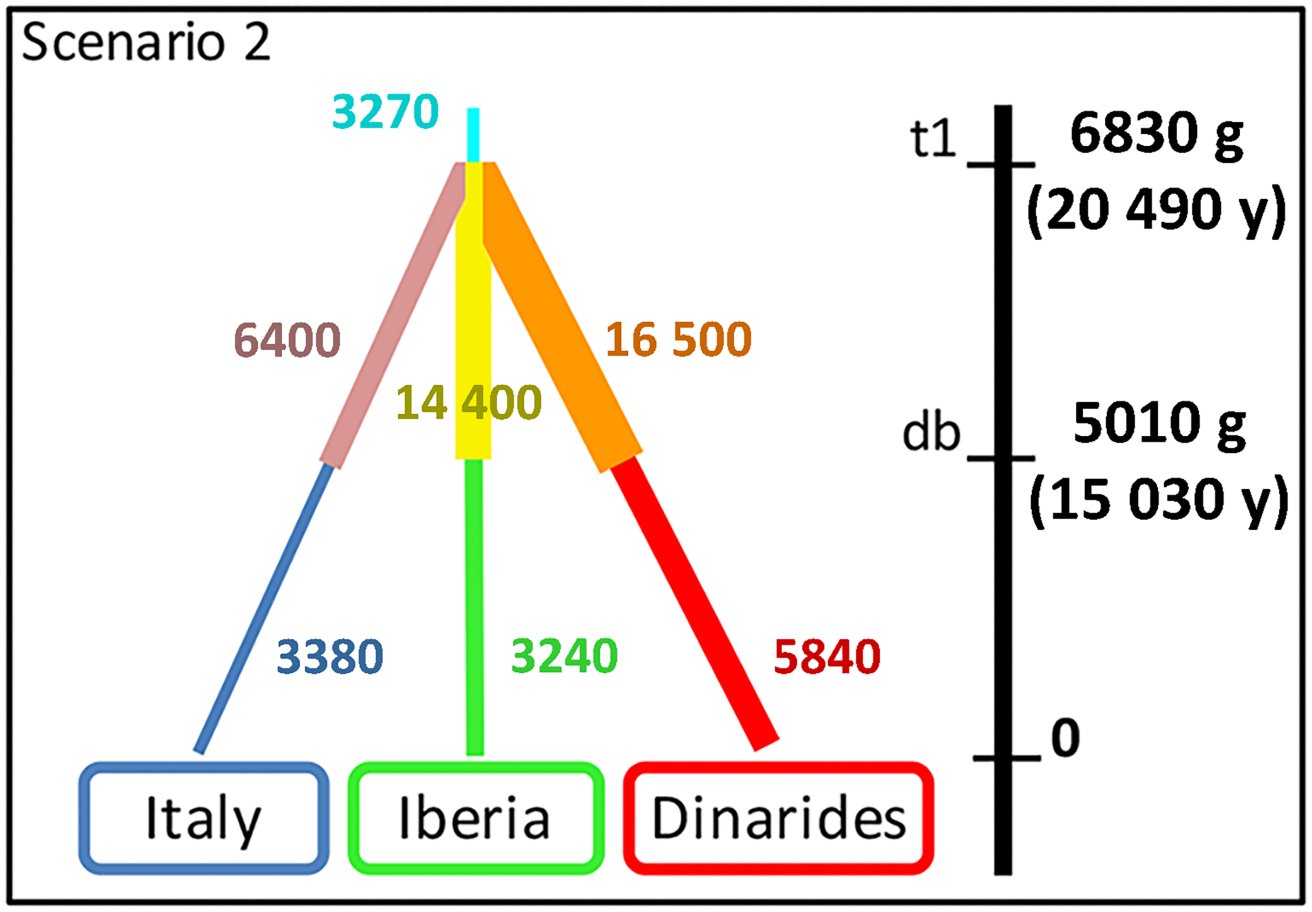
Best scenarios as inferred by Diyabc. Graphical representation of the resulting population sizes and divergence times estimated for the two best simulated scenarios, using a generation time g = 3 years. The width of branches is proportional to the inferred effective population sizes.

The best scenario showed non-significant *P*-values for all the posterior parameters after Bonferroni correction (S7 Table). Under this scenario the median values of the divergence time showed that the three wolf populations have been genetically isolated for the last 6830 generations (5% quantile (q050) = 3240 generations– 95% quantile (q950) = 9600 generations) (Table 3). Assuming a wolf generation time of three years [60,61], the TMRCA of these populations is 20 490 years ago, while their bottlenecks were estimated around 15 030 years ago (S7 Fig; Table 3). The current effective populations sizes (N1, N2 and N3; S5 Fig) were much lower than the corresponding effective populations sizes before the demographic decline (Table 4, Fig 4). In particular, the bottleneck led the Italian wolf population to decline by *c*. 1.9 times while the Iberian and Dinaric populations *c*. 4.4–3.0 times, respectively.

**Table 3:** Original parameter estimation and statistics (median and quantiles) of the posterior distribution for the scenario with the highest posterior probabilities.

Parameters Scenario 2	median	q050	q950
**N1**	3.38E+03	1.50E+03	7.76E+03
**N2**	3.24E+03	7.85E+02	8.28E+03
**N3**	5.48E+03	1.51E+03	9.29E+03
**t1**	6.83E+03	3.42E+03	9.60E+03
**db**	5.01E+03	6.51E+02	9.28E+03
**N1b**	6.40E+03	7.46E+02	2.65E+04
**N2b**	1.44E+04	3.47E+03	2.85E+04
**N3b**	1.65E+04	4.60E+03	2.87E+04
**NA**	3.27E+03	3.55E+02	8.29E+03
**Âμmic_1**	1.72E-04	1.10E-04	3.48E-04
**pmic_1**	1.04E-01	1.00E-01	1.36E-01
**snimic_1**	2.67E-06	5.44E-07	7.53E-06

N1-N2-N3 = Italian-Iberian-Dinaric post-bottleneck effective population sizes; N1b-N2b-N3b = Italian-Iberian-Dinaric pre-bottleneck effective population sizes; NA = effective population size of the starting population; t1 = time of divergence from common ancestor in thousands of generations (3 years per generation in *C*. *lupus*); db = duration of bottleneck; Âμmic_1 = mean mutation rate; pmic_1 = mean coefficient P; snimic_1 = mean SNI rate.

**Table 4:** Size of the bottleneck in four wolf populations as estimated by DIYABC under the best two demographic scenarios described in S5 Fig.

Scenario	Italian Peninsula (N1b/N1)	R	Iberian Peninsula (N2b/N2)	R	Dinaric regions (N3b/N3)	R
2	6400/3380	1.9	14.400/3240	4.4	16.500/5480	3.0

N = post-bottleneck effective population size. Nb = pre-bottleneck effective population size. R = Nb/N ratio.

## Discussion

During the last few centuries large carnivores and ungulates declined in south-western Europe as consequence of deforestation, overhunting and direct persecution [4,62]. In peninsular Italy wolves survived in isolation after protracted range contraction and strong demographic decline. In the first half of the 1970s, during the most recent bottleneck, only *c*. 100 individuals remained in isolated mountain areas in central-southern Italy [14]. Several sources of genetic data concordantly highlighted the consequences of the population bottleneck. Microsatellite markers and genome-wide SNP screenings showed that the Italian wolves have *c*. 33%–42% less autosomal genetic variability than all the other wolf populations in Europe [13,16,52]. An exception is the isolated Iberian wolf population, which also shows low genetic variability [16,63]. Furthermore, uniparental genetic diversity is also very low in the Italian wolves, and until recently, only one mtDNA CR and two Y-chromosome haplotypes were described [20,64]. Inferences from those estimates of genetic variability indicated that wolves might have been isolated in peninsular Italy for thousands of generations, thus ruling out a predominant effect of the most recent anthropogenic bottleneck [13,53]. However, the time of the effective genetic isolation south of the Alps is controversial, with estimates ranging from c. 3000 to 19 000 years depending of the molecular markers (microsatellites, SNPs or entire genomes), or on the assumptions about the effective population size and mutation rates [13,17,53]. The genetic consequences of the population bottlenecks and the origin of the standing genetic variation in the Italian wolves are still not satisfactorily understood. The results of our study confirm that the mtDNA CR haplotype WH19 is not originated via introgressive hybridization with dogs, but instead it was likely already present in the Italian propulation before the 20^th^ century bottleneck [19]. In Bayesian admixture analyses, the autosomal multilocus genotypes of the five samples with haplotype WH19 were entirely assigned to the Italian wolf population (S2 Table). Moreover, we did not find this haplotype in any other wolf population worldwide, nor in dogs. Haplotype WH19 is identical to WH14, except for one SNP at the control region. Closely related to these two haplotypes is haplotype WH17, found in two wolves from Greece. These three haplotypes belong to the same monophyletic mtDNA clade A1 (Fig 3), which includes four other related haplotypes identified in wolves sampled mainly in southern Europe and in the Balkans: Peninsular Italy (haplotypes WH14 and WH19), Greece (WH15, WH17 and WH18), Bulgaria (WH18), Slovenia (WH20), and Poland (WH21), consistent with Pilot et al. [57]. This phylogeographic pattern could represent ancient relic lineages, as indicated by the basal position of clade A in the phylogenetic trees (Fig 3 and S4 Fig). The origin of clade A1 seems extremely recent, dating back to 6800 years ago (95% HDP = 2600–12 400 years), when it coalesced with its sister clade A2. This latter includes the haplotype S14.5k, which was identified in an ancient wolf sample from Switzerland dated at *c*. 14 500 years ago [56], and five haplotypes found only in ancient Scandinavian dog breeds (S3 Table; Fig 3). Clade B can also be divided in two sub-clades, with clade B1 including the haplotypes found in clade 1 from Pilot et al. [57], and clade B2 containing haplotypes that are intermediate between clades 1 and 2 from Pilot et al. [57], consistent with Thalmann et al. [56]. Moreover, our clades A and B coalesced 45 400 years ago (95% HDP = 35 100–57 200 years), coherently with Thalmann et al. [56] and Koblmüller et al. [65], suggesting that the entire mtDNA diversity in extant wolf populations has been generated during the last glaciations (the Würm glaciation in Europe), early Holocene.

From a geographical point of view, the haplotypes of the highly diverse haplogroup B are widespread in Europe, Asia and North America (confirming [57]). In contrast, haplotypes in clade A have more limited distributions centered in southern-central European countries. This pattern, together with archaeological, morphological and genetic findings, suggest that extant Old and New World wolf populations expanded during or right after the last glacial period, balancing the local extinction of ancient wolf ecomorphs [53,56,65–68]. However, it is not known if the turnover of wolf populations has been the consequence of a generalized megafaunal extinction wave due to climate changes or to hunting pressure and/or competition with expanding modern human populations [69–71]. Historical recolonization patterns might have been blurred by the extreme wolf potential to disperse or by recent anthropogenic eradication of many local populations [72]. Samples and genetic data of wolves from Eastern Europe and Asia are still scanty, preventing the reconstruction of the presumably complex population dynamics in those areas. For instances, while Italy is the single area where only haplogroup A is found, the poorly sampled sectors of Carpathian, Balkan and Caucasian regions show the presence of haplotypes from both A and B haplogroups, suggesting that these are (or were) areas of ancient population admixture (this study and [52,57]). Furthermore, the extinction of ancient mtDNA lineages and specialized wolf ecomorphs has been documented in North America, but it is not well described in Europe [67,73,74]. The lack of information on the extent of genetic and phenotypic variation in Paleolithic wolf populations in Eurasia makes the identification of wolf populations and the areas of origin of domesticated dogs difficult [75]. A better comprehension of these phenomena will help to reconstruct the wolf phylogeography in Eurasia that, at present, is still incomplete.

The phylogeographic patterns emerging from the mtDNA data are completed by multivariate and Bayesian analyses of the multilocus autosomal wolf genotypes. Results indicate that the main geographical populations in central and southern Europe are genetically distinct. The Italian and the Iberian wolves were the first ones to cluster separately in both multivariate and Bayesian clustering, confirming Lucchini et al. [13], Stronen et al. [52], and Pilot et al. [16]. Wolves sampled from populations in the eastern countries, and particularily from the Balkan and Carpathian regions show signatures of admixture, supporting previous mtDNA and autosomal SNP findings [16,52]. Recent genomic analyses [17] suggest that the most plausible demographic scenario should assume closely sequential splitting wolf population bottlenecks. ABC simulations indicate that southern European wolf populations (Iberian, Italian and Dinaric) might have split simultaneously at 20 490 and bottlenecked at 15 030 years ago (Table 3; Fig 4). Simulations also showed that all the current southern European wolf effective population sizes are significantly lower than sizes before bottlenecks with the Italian population that declined by *c*. 1.9 and the other populations *c*. 3.0–4.4 times, coherently with inferences from genomic data [53].

## Conclusions

Despite the uncertainty that is usually associated to TMRCA estimates computed from limited number of markers (as in this study), or from limited number of samples (as in the genomic studies published so far [17,53], results concordantly show that currently fragmented wolf populations in south-western Europe were effectively isolated right after the last glacial maximum (*c*. 20 000–16 000 years ago). During this process, the Italian wolf population underwent a sharp demographic decline, which strongly reduced its standing genetic diversity. Thus, the result of the ancient isolation compromised the genetic pool of the Italian wolves long before the most recent anthropogenic bottleneck. However, these findings do not rule out the possibility that a recent demographic decline impoverished even more the genetic variation of this population.

## Supporting information

S1 Fig Delta *K* values [29] obtained in Structure analyses of dog and wolf samples assuming *K* values from 1 to 15. (PDF)

S2 Fig Results of Bayesian clustering analyses of dog and wolf samples obtained by Structure assuming *K* values from 1 to 15. Dog and wolf population samples are shown in the same sequence as in Fig 2: dogs (1), Italian wolves (2), Iberian wolves (3), Dinaric wolves (4), Balkanic wolves (5), Carpathian wolves (6), Baltic wolves (7).
(PDF)

S3 Fig (A) NJ and (B) consensus ML mtDNA phylogenetic trees. Details of clade A are highlighted in the top -left figures.
(PDF)

S4 Fig Phylogenetic tree of concatenated multi-fragment (control-region, ATP6, COIII and ND4) dog and wolf mtDNA haplotypes generated using a Bayesian procedure implemented in MrBayes [41]. A homologous concatenated sequence of *Canis latrans* (DQ480509) is used as an outgroup. Every node shows its posterior probability. Clade A, that includes the Italian wolf haplotypes WH14 and WH19, is highlighted at the top left of the figure.
(PDF)

S5 Fig ABC demographic scenarios and locations of the selected wolf populations. (PDF)

S6 Fig Model checking. Pre-evaluation of scenario-prior combinations; direct and logistic regression comparison methods of the estimated posterior probabilities among scenarios and fit of the selected best scenarios (Sc2 and Sc4) with the observed data. PCA I and II plotted using 10.000 data points.
(PDF)

S7 Fig Prior (red) and posterior (green) density distributions of posterior probability for the selected ABC parameters from scenarios 2 and 4. (PDF)

S1 Table Description of the genotyped autosomal (CFA) microsatellites (STR). (PDF)

S2 Table List of the wolf and dog samples analyzed in this study indicating: The country of origin, taxon, gender, mtDNA haplotypes at ATP6, COIII, ND4, CR and the concatenated multifragment sequences (MF). The individual Bayesian clustering assignments were computed using Structure with *K* = 3, assuming that genotypes could have ancestry in a dog cluster (*q*_*d*_), an Italian wolf cluster (*q*_*wit*_), or un a third wolf cluster including all the other wolves (*q*_*weu*_).
(PDF)

S3 Table List of mtDNA sequences downloaded from the GenBank. For every sample is shown: the accession number, country of origin of the sequenced sample (if available), taxon, dog breed (if available), and clade of memberships (BEAST analysis), and haplotypes at different genic regions.
(PDF)

S4 Table DIYABC prior distributions for demographic parameters and mutation rates. (PDF)

S5 Table Haplotype composition. Detailed composition of the concatenated multifragment haplotypes.
(PDF)

S6 Table Distribution of the concatenated multifragment mtDNA haplotypes in wolves sampled all over the world. Country acronyms: IB = Iberian Peninsula; IT = Italy; HR = Croatia; SL = Slovenia; GR = Greece; BG = Bulgaria; PL = Poland; ES = Estonia; LA = Latvia; FI = Finland; UK = Ukraine; SW = Sweden; IR = Iran; OM = Oman; SA = Saudi Arabia; IS = Israel; IN = India; CH = China; AK = Alaska; CA = Canada; US = USA; MX = Mexico.
(PDF)

S7 Table Model checking results for the best fitting scenario (SC2) based on 1,000 simulated datasets. (PDF)

S1 Appendix (A) Description of laboratory methods with details on primers and PCR profiles for all the genotyped markers and (B) Details on MrBayes and BEAST models. (PDF)

## References

[1] Pereira HM. Rewilding European Landscapes. Pereira HM, Navarro L, editors. Springer International Publishing; 2015.

[2] Crooks KR, Burdett CL, Theobald DM, Rondinini C, Boitani L. Global patterns of fragmentation and connectivity of mammalian carnivore habitat. Philos Trans R Soc B Biol Sci. 2011;366: 2642–2651. 10.1098/rstb.2011.0120 PMC3140740 21844043

[3] Linnell JDC, Swenson JE, Andersen R. Predators and people: conservation of large carnivores is possible at high human densities if management policy is favourable. Anim Conserv. 2001;4: 345–349.

[4] Chapron G, Kaczensky P, Linnell JDC, von Arx M, Huber Đ, Andrén H, et al Recovery of large carnivores in Europe’s modern human-dominated landscapes. Science. 2014;346: 1517–1519. 10.1126/science.1257553 25525247

[5] Nei M, Maruyama T, Chakraborty R. The Bottleneck Effect and genetic variability in Populations. Evolution. 1975;29: 1–10. 28563291 10.1111/j.1558-5646.1975.tb00807.x

[6] Lacy RC. Loss of genetic diversity from managed populations: interacting effects of drift, mutation, immigration, selection, and population subdivision. Conserv Biol. 1987;1: 143–58.

[7] Palstra FP, Fraser DJ. Effective/census population size ratio estimation: a compendium and appraisal. Ecol Evol. 2012; 2357–2365. 10.1002/ece3.329 23139893 PMC3488685

[8] Bertorelle G, Benazzo A, Mona S. ABC as a flexible framework to estimate demography over space and time: some cons, many pros. Mol Ecol. 2010;19: 2609–2615. 10.1111/j.1365-294X.2010.04690.x 20561199

[9] Hoban S, Bertorelle G, Gaggiotti OE. Computer simulations: tools for population and evolutionary genetics. Nat Rev Genet. 2012;13: 110–122. 10.1038/nrg3130 22230817

[10] Lohmueller KE, Albrechtsen A, Li Y, Kim SY, Korneliussen T, Vinckenbosch N, et al Natural selection affects multiple aspects of genetic variation at putatively neutral sites across the human genome. PLoS Genet. 2011;7. 10.1371/journal.pgen.1002326 PMC3192825 22022285

[11] Chapman JR, Nakagawa S, Coltman DW, Slate J, Sheldon BC. A quantitative review of heterozygosity-fitness correlations in animal populations. Mol Ecol. 2009;18: 2746–2765. 10.1111/j.1365-294X.2009.04247.x 19500255

[12] Boitani L. Wolf research and conservation in Italy. Biol Conserv. 1992;61: 1992.

[13] Lucchini V, Galov A, Randi E. Evidence of genetic distinction and long-term population decline in wolves (Canis lupus) in the Italian Apennines. Mol Ecol. 2004;13: 523–536. 14871358 10.1046/j.1365-294x.2004.02077.x

[14] Boitani L. Wolf conservation and recovery In: Mech LD, Boitani L, editors. Wolves: behavior, ecology and conservation. The University of Chicago Press; 2003 pp. 317–340.

[15] Galaverni M, Caniglia R, Fabbri E, Milanesi P, Randi E. One, no one, or one hundred thousand: how many wolves are there currently in Italy? Mammal Res. 2016;61: 13–24.

[16] Pilot M, Dąbrowski MJ, Hayrapetyan V, Yavruyan EG, Kopaliani N, Tsingarska E, et al Genetic variability of the grey wolf Canis lupus in the caucasus in comparison with Europe and the Middle East: Distinct or intermediary population? PLoS One. 2014;9. 10.1371/journal.pone.0093828 PMC3979716 24714198

[17] Silva PM. Historical demography and differentiation of the gray wolf (Canis lupus). Universidade do Porto, Portugal 2016.

[18] Randi E, Lucchini V, Christensen MF, Mucci N, Funk SM, Dolf G, et al Mitochondrial DNA variability in Italian and east European wolves: Detecting the consequences of small population size and hybridization. Conserv Biol. 2000;14: 464–473.

[19] Montana L, Caniglia R, Galaverni M, Fabbri E, Randi E. A new mitochondrial haplotype confirms the distinctiveness of the Italian wolf (Canis lupus) population. Mamm Biol—Zeitschrift für Säugetierkd. Elsevier GmbH; 2017;84: 30–34.

[20] Randi E, Hulva P, Fabbri E, Galaverni M, Galov A, Kusak J, et al Multilocus detection of wolf x dog hybridization in Italy, and guidelines for marker selection. PLoS One. 2014;9. 10.1371/journal.pone.0086409 PMC3899229 24466077

[21] Leonard JA, Echegaray J, Randi E, Vilà C. Impact of hybridization with domestic dogs on the conservation of wild canids In: Gompper ME, editor. Free-Ranging Dogs and Wildlife Conservation. Oxford University Press; 2014 pp. 170–184.

[22] Hindrikson M, Männil P, Ozolins J, Krzywinski A, Saarma U. Bucking the Trend in Wolf-Dog Hybridization: First Evidence from Europe of Hybridization between Female Dogs and Male Wolves. PLoS One. 2012;7: 1–12. 10.1371/journal.pone.0046465 PMC3463576 23056315

[23] Longmire JL, Maltbie M, Baker RJ. Use of”lysis buffer” in DNA isolation and its implication for museum collections. Museum of Texas Tech University; 1997.

[24] de Groot GA, Nowak C, Skrbinšek T, Andersen LW, Aspi J, Fumagalli L, et al Decades of population genetic research reveal the need for harmonization of molecular markers: The grey wolf Canis lupus as a case study. Mamm Rev. 2015; 1–16.

[25] Weir BS, Cockerham CC. Estimating F-statistics for the analysis of population structure. Evolution. 1984;1: 1358–1370. 10.1111/j.1558-5646.1984.tb05657.x 28563791

[26] Peakall R, Smouse PE. GenAlEx 6.5: genetic analysis in Excel. Population genetic software for teaching and research—an update. Bioinformatics. 2012;28: 2537–2539. 10.1093/bioinformatics/bts460 22820204 PMC3463245

[27] Belkhir K, Borsa P, Chikhi L, Raufaste N, Bonhomme F. GENETIX 4.05, Population genetics software for Windows TM. Université de Montpellier II. Montpellier. 2004. 2004. p. 2004.

[28] Jombart T, Ahmed I. adegenet 1.3–1: new tools for the analysis of genome-wide SNP data. Bioinformatics. 2011;27: 3070–3071. 10.1093/bioinformatics/btr521 21926124 PMC3198581

[29] Pritchard JK, Stephens M, Donnelly P. Inference of Population Structure Using Multilocus Genotype Data. Genetics. 2000;155: 945–959. 10835412 10.1093/genetics/155.2.945 PMC1461096

[30] Hubisz MJ, Falush D, Stephens M, Pritchard JK. Inferring weak population structure with the assistance of sample group information. Mol Ecol Resour. 2009;9: 1322–1332. 10.1111/j.1755-0998.2009.02591.x 21564903 PMC3518025

[31] Evanno G, Regnaut S, Goudet J. Detecting the number of clusters of individuals using the software STRUCTURE: a simulation study. Mol Ecol. 2005;14: 2611–2620. 10.1111/j.1365-294X.2005.02553.x 15969739

[32] Jakobsson M, Rosenberg NA. CLUMPP: a cluster matching and permutation program for dealing with label switching and multimodality in analysis of population structure. Bioinformatics. 2007;23: 1801–1806. 10.1093/bioinformatics/btm233 17485429

[33] Fabbri E, Caniglia R, Kusak J, Galov A, Gomerčić T, Arbanasić H, et al Genetic structure of expanding wolf (Canis lupus) populations in Italy and Croatia, and the early steps of the recolonization of the Eastern Alps. Mamm Biol—Zeitschrift für Säugetierkd. 2014;79: 138–148.

[34] Björnerfeldt S, Webster MT, Vilà C. Relaxation of selective constraint on dog mitochondrial DNA following domestication. Genome Res. 2006;16: 990–994. 10.1101/gr.5117706 16809672 PMC1524871

[35] Librado P, Rozas J. DnaSP v5: a software for comprehensive analysis of DNA polymorphism data. Bioinformatics. 2009;25: 1451–1452. 10.1093/bioinformatics/btp187 19346325

[36] Nei M, Li W-H. Mathematical model for studying genetic variation in terms of restriction endonucleases. Proc Natl Acad Sci USA. 1979;76: 5269–5273. 291943 10.1073/pnas.76.10.5269 PMC413122

[37] Saitou N, Nei M. The Neighbor-joining Method: A New Method for Reconstructing Phylogenetic Trees. Mol Biol Evol. 1987;4: 406–425. 3447015 10.1093/oxfordjournals.molbev.a040454

[38] Felsenstein J. Evolutionary Trees from DNA Sequences: A Maximum Likelihood Approach. J Mol Evol. 1981;17: 368–376. 7288891 10.1007/BF01734359

[39] Rannala BH, Yang Z. Probability distribution of molecular evolutionary trees: A new method of phylogenetic inference. J Mol Evol. 1996;43: 304–311. 8703097 10.1007/BF02338839

[40] Swofford DL. PAUP* Phylogenetic Analysis Using Parsimony (*and other methods). Sinauer Associates, Sunderland; 2003 p. 2003.

[41] Ronquist F, Teslenko M, van der Mark P, Ayres DL, Darling A, Höhna S, et al MrBayes 3.2: Efficient Bayesian Phylogenetic Inference and Model Choice Across a Large Model Space. Syst Biol. 2012;61: 539–542. 10.1093/sysbio/sys029 22357727 PMC3329765

[42] Posada D, Crandall KA. MODELTEST: testing the model of DNA substitution. Bioinformatics. 1998;14: 817–818. 9918953 10.1093/bioinformatics/14.9.817

[43] Akaike H. Information theory as an extension of the maximum likelihood principle. In: Petrov BN, Csaki F, editors. Second International Symposium on Information Theory. 1973. pp. 267–281.

[44] Felsenstein J. Confidence Limits on Phylogenies: An Approach Using the Bootstrap. Evolution. 1985;39: 783–791. 28561359 10.1111/j.1558-5646.1985.tb00420.x

[45] Lanfear R, Calcott B, Ho SYW, Guindon S. PartitionFinder: Combined Selection of Partitioning Schemes and Substitution Models for Phylogenetic Analyses. Mol Biol Evol. 2012;29: 1695–1701. 10.1093/molbev/mss020 22319168

[46] Larget B, Simon DL. Markov Chain Monte Carlo Algorithms for the Bayesian Analysis of Phylogenetic Trees. Mol Biol Evol. 1999;16: 750–759.

[47] Bouckaert R, Heled J, Kühnert D, Vaughan T, Wu C-H, Xie D, et al BEAST 2: a software platform for bayesian evolutionary analysis. PLoS Comput Biol. 2014;10: 1–6. 10.1371/journal.pcbi.1003537 PMC3985171 24722319

[48] Heled J, Drummond AJ. Bayesian inference of population size history from multiple loci. BMC Evol Biol. 2008;8: 289 10.1186/1471-2148-8-289 18947398 PMC2636790

[49] Beaumont MA, Zhang W, Balding DJ. Approximate Bayesian Computation in Population Genetics. Genetics. 2002;162: 2025–2035. 12524368 10.1093/genetics/162.4.2025 PMC1462356

[50] Cornuet J-M, Pudlo P, Veyssier J, Dehne-Garcia A, Gautier M, Leblois R, et al DIYABC v2.0: a software to make approximate Bayesian computation inferences about population history using single nucleotide polymorphism, DNA sequence and microsatellite data. Bioinformatics. 2014;30: 1187–1189. 10.1093/bioinformatics/btt763 24389659

[51] Hindrikson M, Remm J, Männil P, Ozolins J, Tammeleht E, Saarma U. Spatial Genetic Analyses Reveal Cryptic Population Structure and Migration Patterns in a Continuously Harvested Grey Wolf (Canis lupus) Population in North-Eastern Europe. PLoS One. 2013;8: 1–12. 10.1371/journal.pone.0075765 PMC3777892 24069446

[52] Stronen AV, Jędrzejewska B, Pertoldi C, Demontis D, Randi E, Niedziałkowska M, et al North-South Differentiation and a Region of High Diversity in European Wolves (Canis lupus). PLoS One. 2013;8: 1–9. 10.1371/journal.pone.0076454 PMC3795770 24146871

[53] Fan Z, Silva P, Gronau I, Wang S, Armero AS, Schweizer RM, et al Worldwide patterns of genomic variation and admixture in gray wolves. Genome Res. 2016;26: 1–11. 10.1101/gr.197517.115 PMC4728369 26680994

[54] Cornuet J-M, Ravigné V, Estoup A. Inference on population history and model checking using DNA sequence and microsatellite data with the software DIYABC (v1.0). BMC Bioinformatics. 2010;11. 10.1186/1471-2105-11-401 PMC2919520 20667077

[55] Boggiano F, Ciofi C, Boitani L, Formia A, Grottoli L, Natali C, et al Detection of an East European wolf haplotype puzzles mitochondrial DNA monomorphism of the Italian wolf population. Mamm Biol—Zeitschrift für Säugetierkd. Elsevier GmbH; 2013;78: 374–378.

[56] Thalmann O, Shapiro B, Cui P, Schuenemann VJ, Sawyer SK, Greenfield DL, et al Complete Mitochondrial Genomes of Ancient Canids Suggest a European Origin of Domestic Dogs. Science. 2013;342: 871–874. 10.1126/science.1243650 24233726

[57] Pilot M, Branicki W, Jędrzejewski W, Goszczyński J, Jędrzejewska B, Dykyy I, et al Phylogeographic history of grey wolves in Europe. BMC Evol Biol. 2010;10. 10.1186/1471-2148-10-104 PMC2873414 20409299

[58] Savolainen P, Zhang Y, Luo J, Lundeberg J, Leitner T. Genetic Evidence for an East Asian Origin of Domestic Dogs. Science. 2002;1610: 1610–1613. 10.1126/science.1073906 12446907

[59] Vilà C, Amorim IR, Leonard JA, Posada D, Castroviejo J, Petrucci-Fonseca F, et al Mitochondrial DNA phylogeography and population history of the grey wolf Canis lupus. Mol Ecol. 1999;8: 2089–2103. 10632860 10.1046/j.1365-294x.1999.00825.x

[60] Skoglund P, Götherström A, Jakobsson M. Estimation of Population Divergence Times from Non- Overlapping Genomic Sequences: Examples from Dogs and Wolves. Mol Biol Evol. 2011;28: 1505–1517. 10.1093/molbev/msq342 21177316

[61] Mech LD, Seal US. Premature reproductive activity in wild wolves. J Mammal. 1987;68: 871–873.

[62] Hindrikson M, Remm J, Pilot M, Godinho R, Stronen AV, Baltrūnaitė L, et al Wolf population genetics in Europe: a systematic review, meta-analysis and suggestions for conservation and management. Biol Rev. 2016; 10.1111/brv.12298 27682639

[63] Sastre N, Vilà C, Salinas M, Bologov V V, Urios V, Sánchez A, et al Signatures of demographic bottlenecks in European wolf populations. Conserv Genet. 2011;12: 701–712.

[64] Caniglia R, Fabbri E, Galaverni M, Milanesi P, Randi E. Noninvasive sampling and genetic variability, pack structure, and dynamics in an expanding wolf population. J Mammal. 2014;95: 41–59.

[65] Koblmüller S, Vilà C, Lorente-Galdos B, Dabad M, Ramirez O, Marques-Bonet T, et al Whole mitochondrial genomes illuminate ancient intercontinental dispersals of grey wolves (Canis lupus). J Biogeogr. 2016; 1–11.

[66] Freedman AH, Gronau I, Schweizer RM, Ortega-Del Vecchyo D, Han E, Silva PM, et al Genome Sequencing Highlights the Dynamic Early History of Dogs. PLoS Genet. 2014;10. 10.1371/journal.pgen.1004016 PMC3894170 24453982

[67] Leonard JA, Vilà C, Fox-Dobbs K, Koch PL, Wayne RK, Van Valkenburgh B. Megafaunal Extinctions and the Disappearance of a Specialized Wolf Ecomorph. Curr Biol. 2007;17: 1146–1150. 10.1016/j.cub.2007.05.072 17583509

[68] Flower LOH, Schreve DC. An investigation of palaeodietary variability in European Pleistocene canids. Quat Sci Rev. Elsevier Ltd; 2014;96: 188–203.

[69] Leonard JA. Ecology drives evolution in grey wolves. Evol Ecol Res. 2014;16: 461–473.

[70] Sandom C, Faurby S, Sandel B, Svenning J-C. Global late Quaternary megafauna extinctions linked to humans, not climate change. Proc R Soc B Biol Sci. 2014;281. 10.1098/rspb.2013.3254 PMC4071532 24898370

[71] Bartlett LJ, Williams DR, Prescott GW, Balmford A, Green RE, Eriksson A, et al Robustness despite uncertainty: regional climate data reveal the dominant role of humans in explaining global extinctions of Late Quaternary megafauna. Ecography (Cop). 2015; 1–10.

[72] Randi E. Genetics and conservation of wolves Canis lupus in Europe. Mamm Rev. 2011;41: 99–111.

[73] Leonard JA, Vilà C, Wayne RK. Legacy lost: genetic variability and population size of extirpated US grey wolves (Canis lupus). Mol Ecol. 2005;14: 9–17. 10.1111/j.1365-294X.2004.02389.x 15643947

[74] Perri A. A wolf in dog’s clothing: Initial dog domestication and Pleistocene wolf variation. J Archaeol Sci. 2016;68: 1–4.

[75] Morey DF. In search of Paleolithic dogs: A quest with mixed results. J Archaeol Sci. 2014;52: 300–307.

