# Supplementary figures and images for: Combining phylogenetic and demographic inferences to assess the origin of the genetic diversity in an isolated wolf population
Source: PLoS One. 2017 May 10;12(5):e0176560. doi: 10.1371/journal.pone.0176560 (PMC5425034; doi:10.1371/journal.pone.0176560)

Supplementary Figure S1.

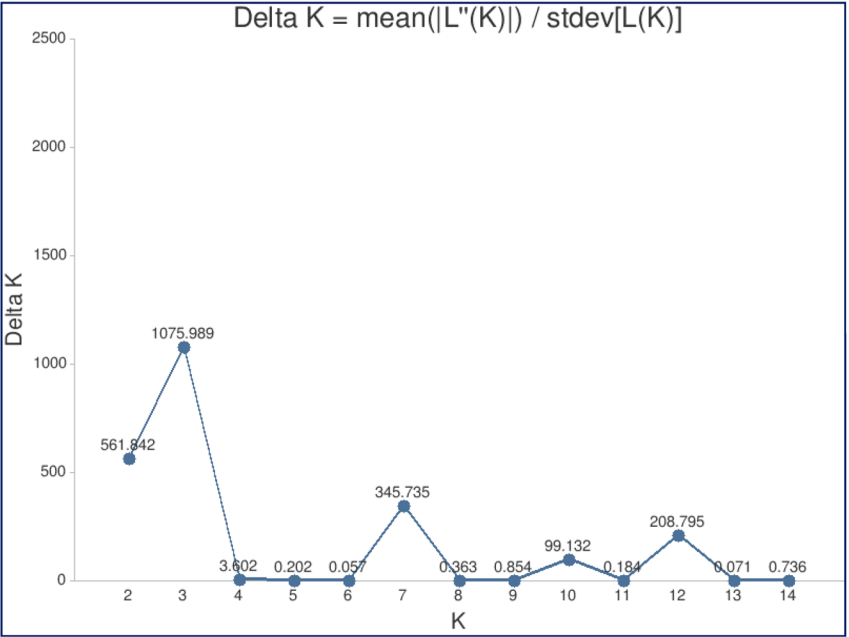

Supplement: S1 Fig — (PDF) [file pone.0176560.s001.pdf]

Supplementary Figure S2.

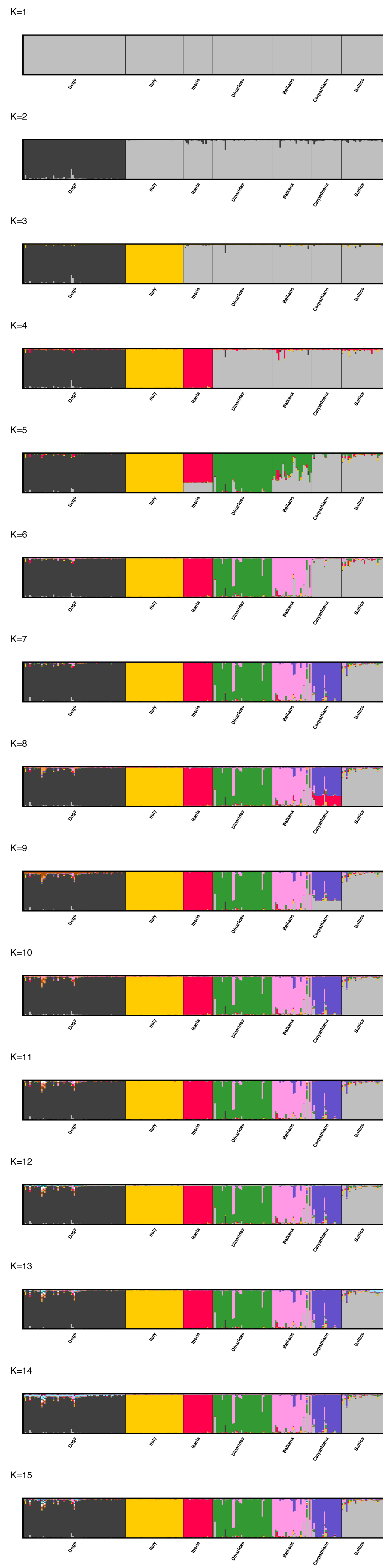

Division of runs by mode:

|      |          |
|------|----------|
| K=1  | 4/4      |
| K=2  | 4/4      |
| K=3  | 5/5      |
| K=4  | 2/4, 2/4 |
| K=5  | 4/4      |
| K=6  | 2/4, 2/4 |
| K=7  | 4/4      |
| K=8  | 4/4      |
| K=9  | 4/4      |
| K=10 | 4/4      |
| K=11 | 4/4      |
| K=12 | 4/4      |
| K=13 | 4/4      |
| K=14 | 4/4      |
| K=15 | 4/4      |

Supplement: S2 Fig — Dog and wolf population samples are shown in the same sequence as in Fig 2: dogs (1), Italian wolves (2), Iberian wolves (3), Dinaric wolves (4), Balkanic wolves (5), Carpathian wolves (6), Baltic wolves (7). (PDF) [file pone.0176560.s002.pdf]

# Neighbor-Joining

## Clade A

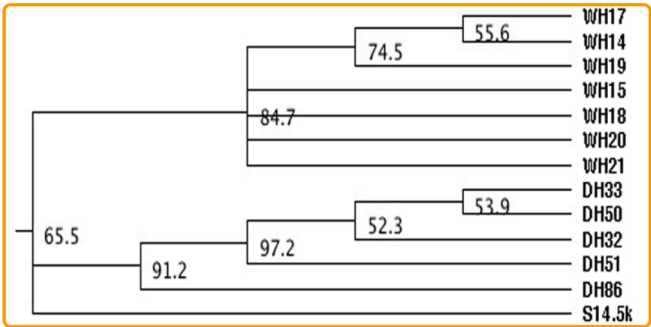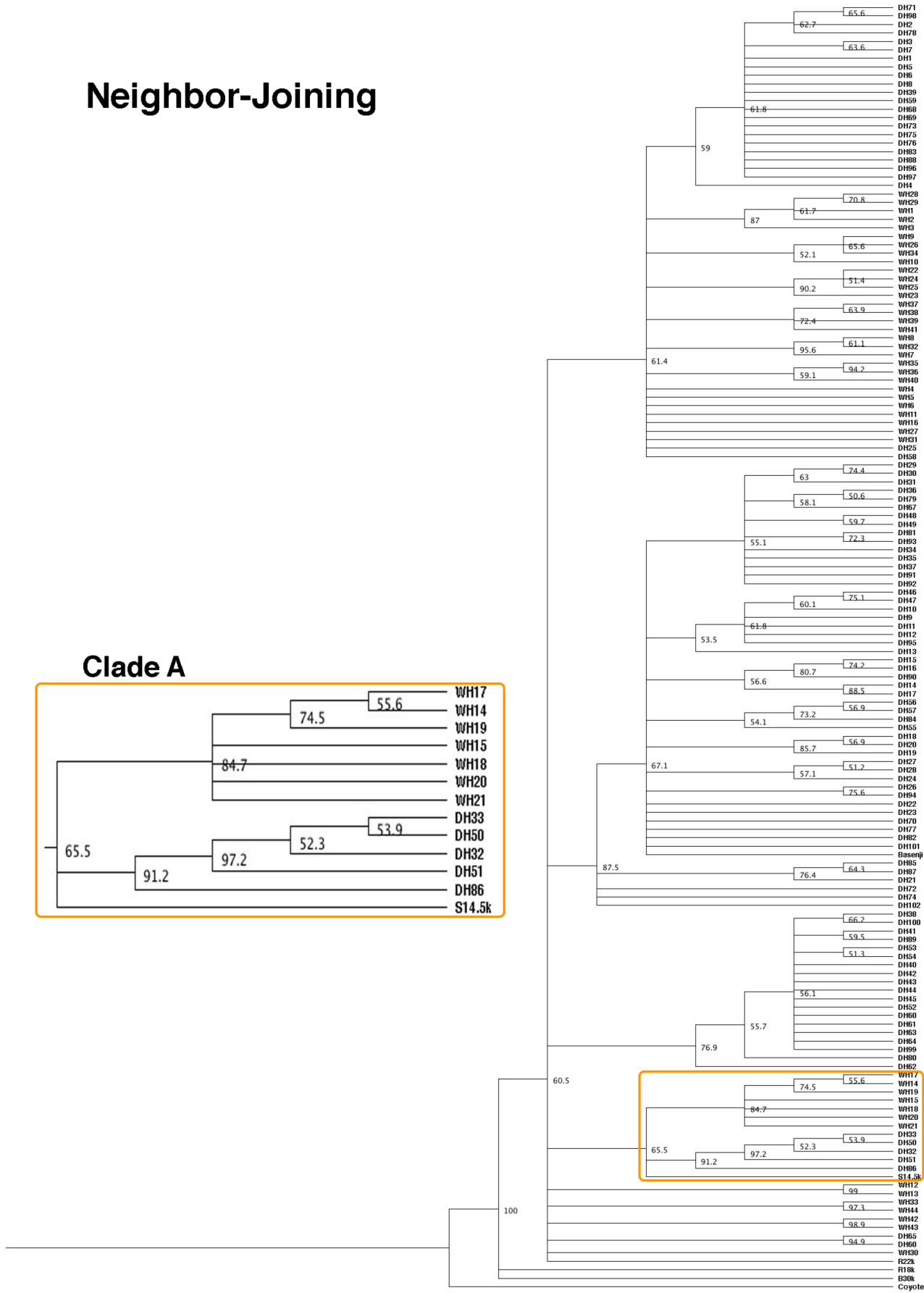

# Maximum Likelihood

## Clade A

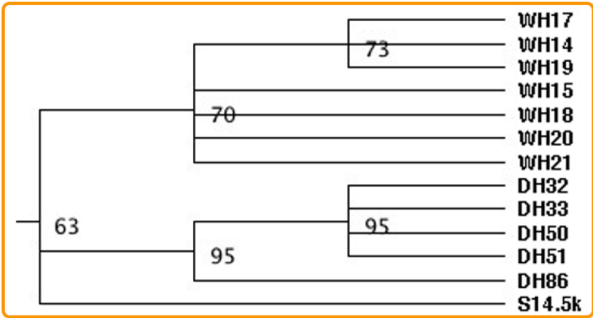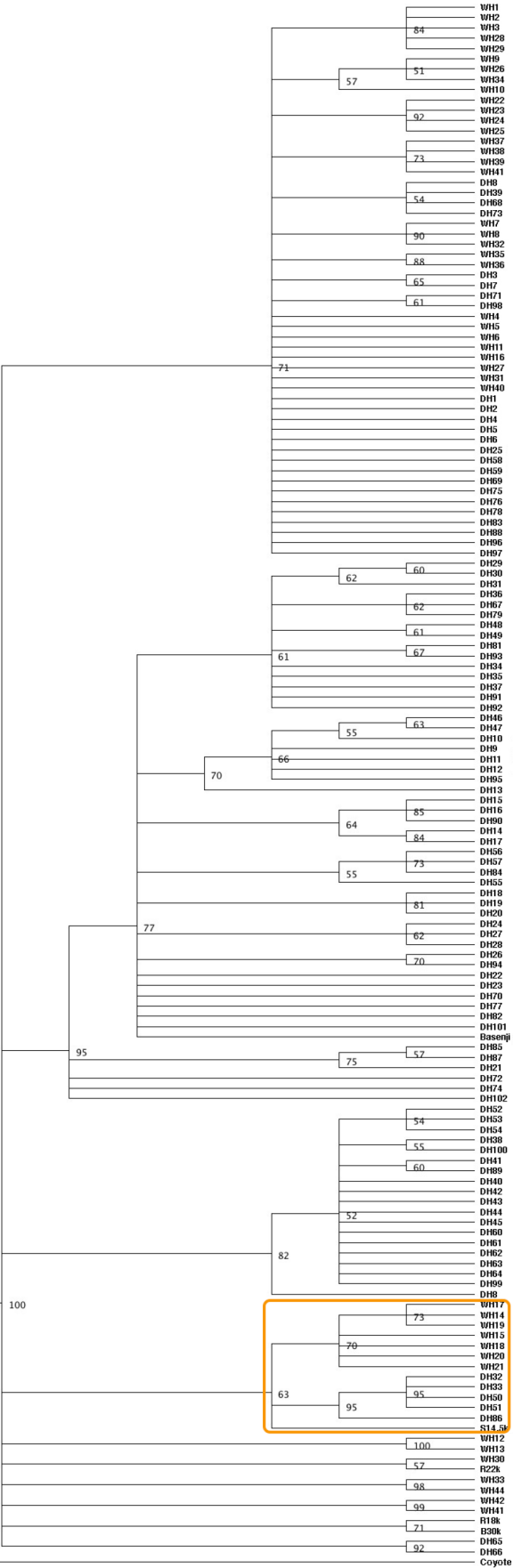

Supplement: S3 Fig — Details of clade A are highlighted in the top -left figures. (PDF) [file pone.0176560.s003.pdf]

Clade A

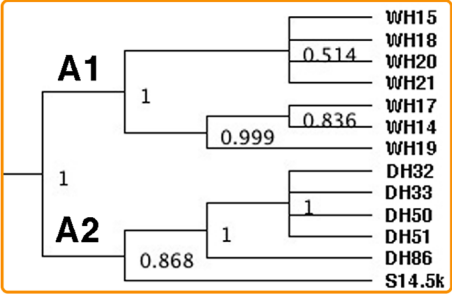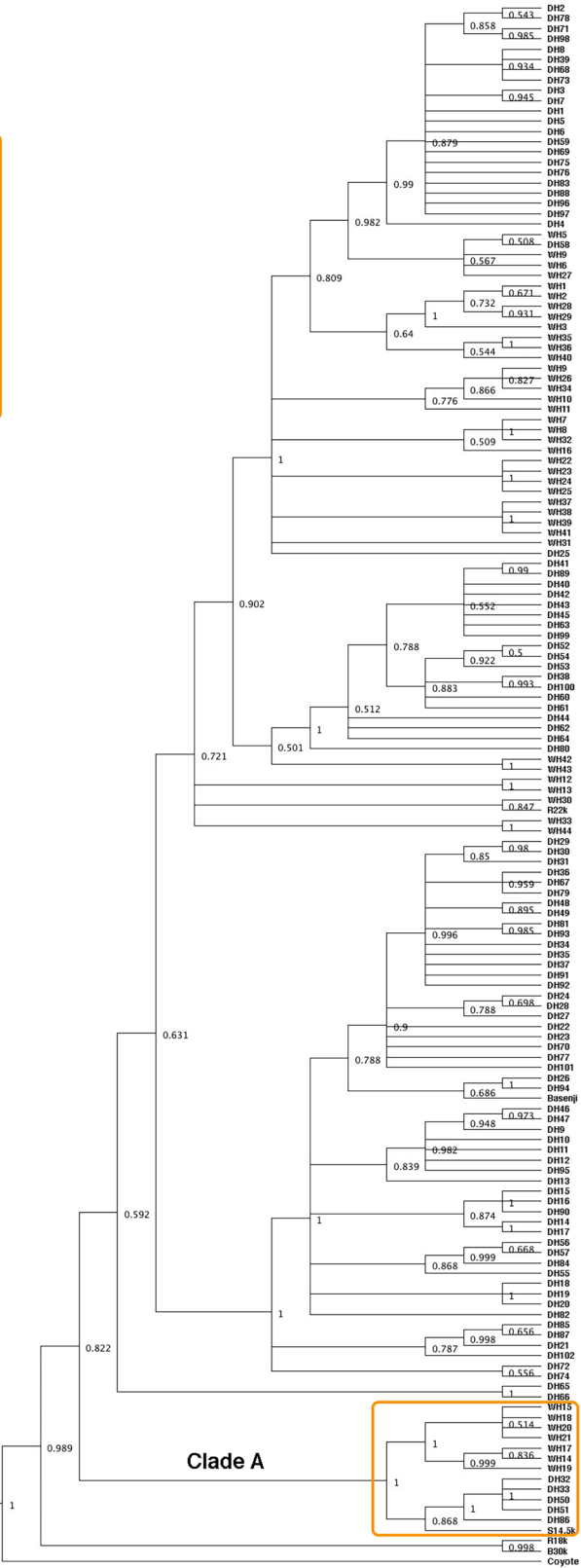

Supplement: S4 Fig — A homologous concatenated sequence of Canis latrans (DQ480509) is used as an outgroup. Every node shows its posterior probability. Clade A, that includes the Italian wolf haplotypes WH14 and WH19, is highlighted at the top left of the figure. (PDF) [file pone.0176560.s004.pdf]

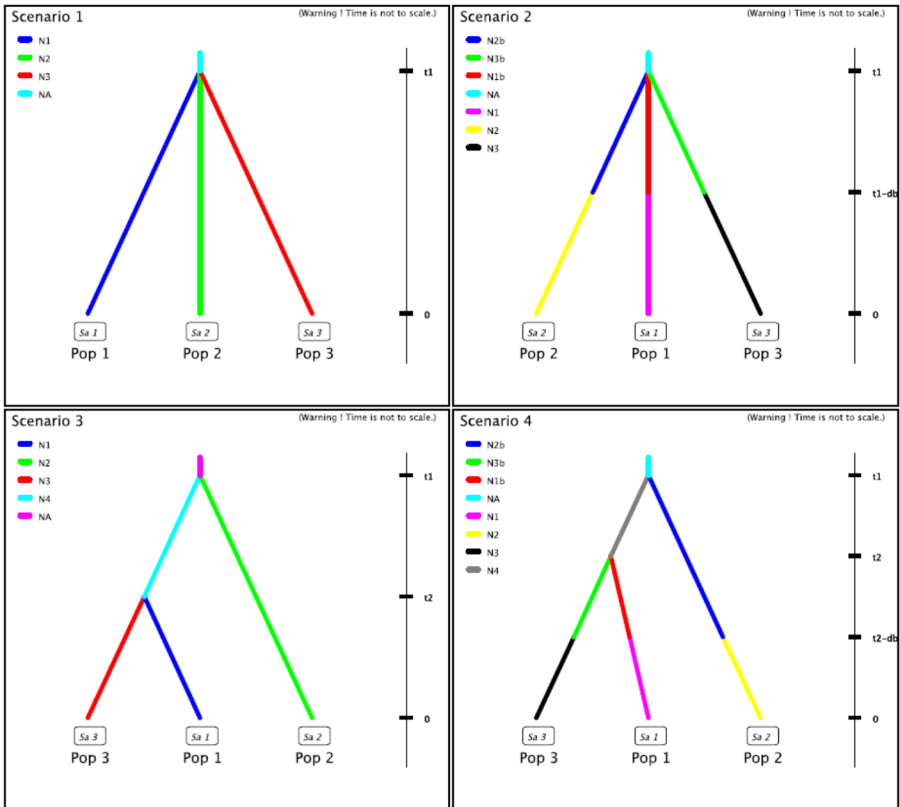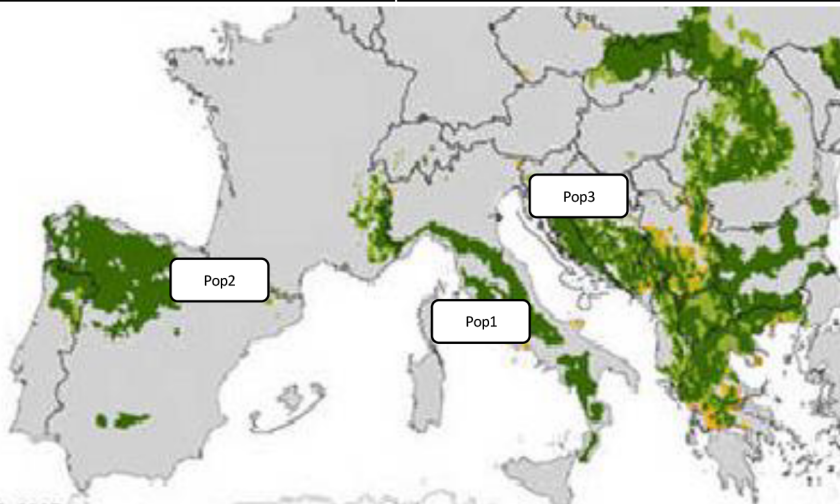

Supplement: S5 Fig — (PDF) [file pone.0176560.s005.pdf]

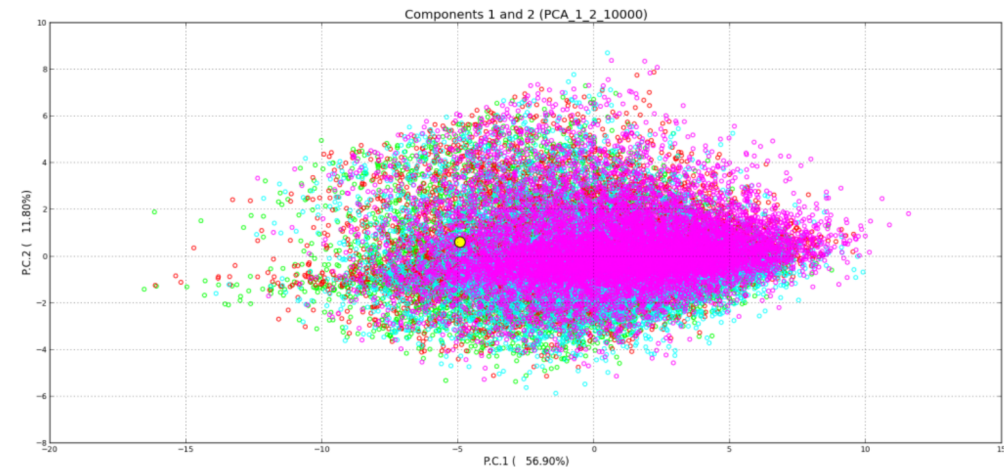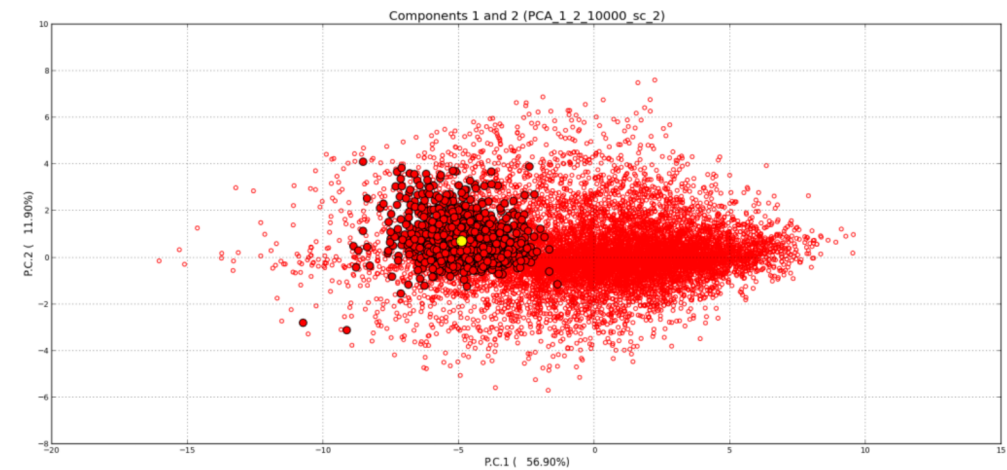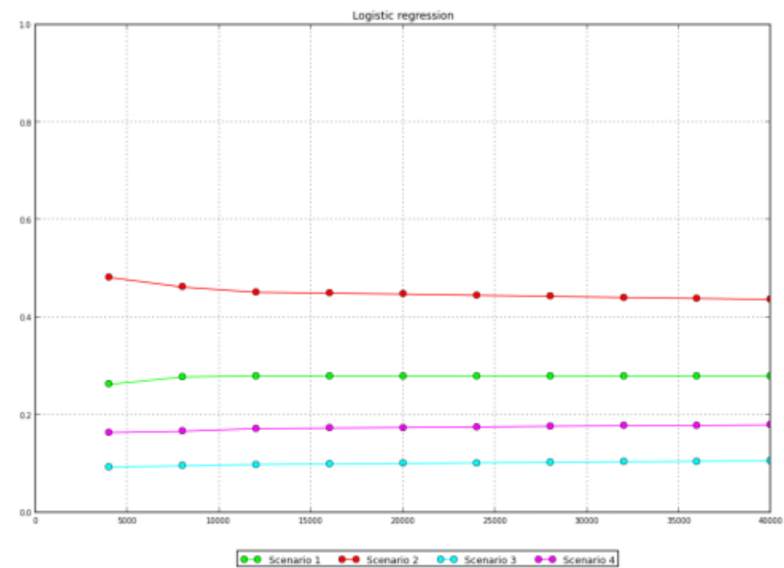

Supplement: S6 Fig — Pre-evaluation of scenario-prior combinations; direct and logistic regression comparison methods of the estimated posterior probabilities among scenarios and fit of the selected best scenarios (Sc2 and Sc4) with the observed data. PCA I and II plotted using 10.000 data points. (PDF) [file pone.0176560.s006.pdf]

N1 [3.38e+03]

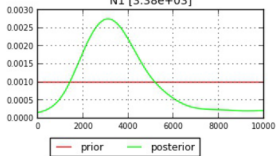

N2 [3.24e+03]

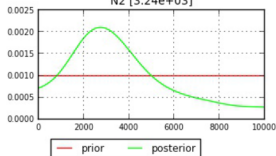

N3 [5.48e+03]

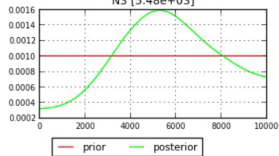

N1b [6.40e+03]

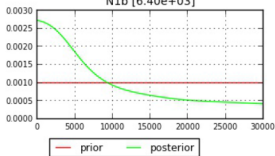

N2b [1.44e+04]

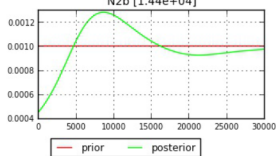

N3b [1.65e+04]

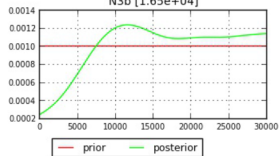

NA [3.27e+03]

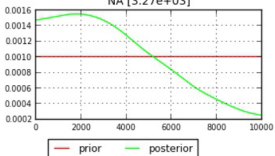

db [5.01e+03]

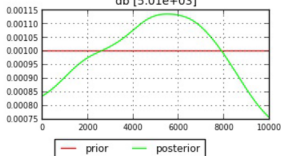

t1 [6.83e+03]

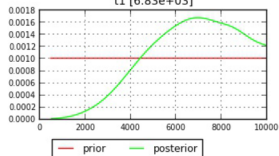

Supplement: S7 Fig — (PDF) [file pone.0176560.s007.pdf]
