# Supplementary material for: Combining phylogenetic and demographic inferences to assess the origin of the genetic diversity in an isolated wolf population
Source: PLoS One. 2017 May 10;12(5):e0176560. doi: 10.1371/journal.pone.0176560 (PMC5425034; doi:10.1371/journal.pone.0176560)
Supplement: S2 Table — The individual Bayesian clustering assignments were computed using Structure with K = 3, assuming that genotypes could have ancestry in a dog cluster (qd), an Italian wolf cluster (qwit), or un a third wolf cluster including all the other wolves (qweu). (PDF) [file pone.0176560.s009.pdf]

S2 Table. List of the wolf and dog samples analyzed in this study indicating: the country of origin, taxon, gender, mtDNA haplotypes at ATP6, COIII, ND4, CR and the concatenated multifragment sequences (MF). The individual Bayesian clustering assignments were computed using STRUCTURE with $K = 3$, assuming that genotypes could have ancestry in a dog cluster (q_d), an Italian wolf cluster (q_{wit}), or un a third wolf cluster including all the other wolves (q_{weu}).

Sample	Country	Taxon	Gender	ATP6	COIII	ND4	CR	MF	q_{weu}	q_{wit}	q_d
**W1667F**	Italy	Wolf	F	A3	C3	N5	W14	**WH14**	*0,001*	*0,998*	*0,001*
**W1860M**	Italy	Wolf	M	A3	C3	N5	W14	**WH14**	*0,001*	*0,998*	*0,001*
**W1088F**	Italy	Wolf	F	A3	C3	N5	W14	**WH14**	*0,001*	*0,998*	*0,001*
**W1452M**	Italy	Wolf	M	A3	C3	N5	W14	**WH14**	*0,001*	*0,998*	*0,001*
**W1435M**	Italy	Wolf	M	A3	C3	N5	W14	**WH14**	*0,001*	*0,998*	*0,001*
**W589M**	Italy	Wolf	M	A3	C3	N5	W14	**WH14**	*0,001*	*0,998*	*0,001*
**W590M**	Italy	Wolf	M	A3	C3	N5	W14	**WH14**	*0,001*	*0,998*	*0,001*
**W028M**	Italy	Wolf	M	A3	C3	N5	W14	**WH14**	*0,002*	*0,997*	*0,001*
**W050M**	Italy	Wolf	M	A3	C3	N5	W14	**WH14**	*0,001*	*0,998*	*0,001*
**W1873M**	Italy	Wolf	M	A3	C3	N5	W14	**WH14**	*0,002*	*0,997*	*0,001*
**W1875M**	Italy	Wolf	M	A3	C3	N5	W14	**WH14**	*0,002*	*0,997*	*0,001*
**W1908F**	Italy	Wolf	F	A3	C3	N5	W14	**WH14**	*0,001*	*0,998*	*0,001*
**W1868M**	Italy	Wolf	M	A3	C3	N5	W14	**WH14**	*0,001*	*0,998*	*0,001*
**W1913F**	Italy	Wolf	F	A3	C3	N5	W14	**WH14**	*0,001*	*0,998*	*0,001*
**W1648M**	Italy	Wolf	M	A3	C3	N5	W14	**WH14**	*0,001*	*0,998*	*0,001*
**W1722F**	Italy	Wolf	F	A3	C3	N5	W14	**WH14**	*0,001*	*0,998*	*0,001*
**W1727M**	Italy	Wolf	M	A3	C3	N5	W14	**WH14**	*0,001*	*0,998*	*0,001*
**W1870F**	Italy	Wolf	F	A3	C3	N5	W14	**WH14**	*0,001*	*0,998*	*0,001*
**W1872F**	Italy	Wolf	F	A3	C3	N5	W14	**WH14**	*0,002*	*0,997*	*0,001*
**W1123M**	Italy	Wolf	M	A3	C3	N5	W14	**WH14**	*0,001*	*0,998*	*0,001*
**W1454F**	Italy	Wolf	F	A3	C3	N5	W14	**WH14**	*0,003*	*0,996*	*0,001*
**W1724M**	Italy	Wolf	M	A3	C3	N5	W14	**WH14**	*0,001*	*0,998*	*0,001*
**W1666F**	Italy	Wolf	F	A3	C3	N5	W14	**WH14**	*0,003*	*0,996*	*0,001*
**W027F**	Italy	Wolf	F	A3	C3	N5	W14	**WH14**	*0,001*	*0,998*	*0,001*
**W040M**	Italy	Wolf	M	A3	C3	N5	W14	**WH14**	*0,002*	*0,997*	*0,001*
**W058F**	Italy	Wolf	F	A3	C3	N5	W14	**WH14**	*0,001*	*0,998*	*0,001*
**W1661M**	Italy	Wolf	M	A3	C3	N5	W14	**WH14**	*0,001*	*0,998*	*0,001*
**W1595F**	Italy	Wolf	F	A3	C3	N5	W14	**WH14**	*0,001*	*0,998*	*0,001*
**W1874F**	Italy	Wolf	F	A3	C3	N5	W14	**WH14**	*0,003*	*0,996*	*0,001*
**W1890M**	Italy	Wolf	M	A3	C3	N5	W14	**WH14**	*0,003*	*0,995*	*0,002*
**W1891M**	Italy	Wolf	M	A3	C3	N5	W14	**WH14**	*0,005*	*0,993*	*0,002*
**W1892M**	Italy	Wolf	M	A3	C3	N5	W14	**WH14**	*0,001*	*0,998*	*0,001*
**W1893M**	Italy	Wolf	M	A3	C3	N5	W14	**WH14**	*0,002*	*0,996*	*0,002*
**W1894M**	Italy	Wolf	M	A3	C3	N5	W14	**WH14**	*0,003*	*0,996*	*0,001*
**W943M**	Italy	Wolf	M	A3	C3	N5	W16	**WH19**	*0,006*	*0,993*	*0,001*
**W1223F**	Italy	Wolf	F	A3	C3	N5	W16	**WH19**	*0,001*	*0,998*	*0,001*
**H1122M**	Italy	Wolf	M	A3	C3	N5	W16	**WH19**	*0,003*	*0,996*	*0,001*

**W1816M**	Italy	Wolf	M	A3	C3	N5	W16	**WH19**	*0,001*	*0,998*	*0,001*
**W1906M**	Italy	Wolf	M	A3	C3	N5	W16	**WH19**	*0,001*	*0,998*	*0,001*
**WIP090**	Iberian P.	Wolf	M	A1	C4	N2	W21	**WH25**	*0,977*	*0,021*	*0,002*
**WIP093**	Iberian P.	Wolf	M	A1	C4	N2	W20	**WH23**	*0,915*	*0,068*	*0,016*
**WIP125**	Iberian P.	Wolf	M	A1	C4	N2	W21	**WH25**	*0,973*	*0,013*	*0,014*
**WIP213**	Iberian P.	Wolf	F	A1	C4	N9	W20	**WH24**	*0,972*	*0,002*	*0,026*
**WIP315**	Iberian P.	Wolf	M	A1	C4	N2	W19	**WH22**	*0,991*	*0,006*	*0,003*
**WIP353**	Iberian P.	Wolf	F	A1	C4	N9	W20	**WH24**	*0,988*	*0,005*	*0,007*
**WIP359**	Iberian P.	Wolf	F	A1	C4	N9	W20	**WH24**	*0,995*	*0,003*	*0,002*
**WIP361**	Iberian P.	Wolf	F	A1	C4	N2	W19	**WH22**	*0,995*	*0,003*	*0,002*
**WIP363**	Iberian P.	Wolf	F	A1	C4	N2	W19	**WH22**	*0,971*	*0,027*	*0,002*
**WIP366**	Iberian P.	Wolf	M	A1	C4	N2	W20	**WH23**	*0,994*	*0,003*	*0,003*
**WIP377**	Iberian P.	Wolf	F	A1	C4	N2	W20	**WH23**	*0,984*	*0,009*	*0,007*
**WIP381**	Iberian P.	Wolf	F	A1	C4	N2	W19	**WH22**	*0,986*	*0,01*	*0,004*
**WIP392**	Iberian P.	Wolf	F	A1	C4	N2	W21	**WH25**	*0,991*	*0,001*	*0,008*
**WIP402**	Iberian P.	Wolf	F	A1	C4	N2	W21	**WH25**	*0,982*	*0,003*	*0,015*
**WIP474**	Iberian P.	Wolf	F	A1	C1	N1	W24	**WH29**	*0,988*	*0,011*	*0,001*
**WIP499**	Iberian P.	Wolf	M	A1	C4	N2	W20	**WH23**	*0,982*	*0,003*	*0,015*
**WIP514**	Iberian P.	Wolf	M	A1	C4	N2	W19	**WH22**	*0,986*	*0,012*	*0,002*
**WIP543**	Iberian P.	Wolf	M	A1	C4	N9	W20	**WH24**	*0,973*	*0,025*	*0,002*
**WIP544**	Iberian P.	Wolf	F	A1	C4	N2	W19	**WH22**	*0,993*	*0,004*	*0,003*
**WIP552**	Iberian P.	Wolf	F	A1	C4	N2	W19	**WH22**	*0,99*	*0,004*	*0,006*
**WSLO_052Y**	Slovenia	Wolf	F	A3	C3	N4	W17	**WH20**	*0,996*	*0,002*	*0,002*
**WSLO_053Y**	Slovenia	Wolf	F	A1	C1	N1	W3	**WH3**	*0,991*	*0,002*	*0,007*
**WSLO_057Y**	Slovenia	Wolf	F	A1	C1	N1	W3	**WH3**	*0,995*	*0,003*	*0,002*
**WSLO_070Y**	Slovenia	Wolf	F	A1	C1	N1	W3	**WH3**	*0,996*	*0,002*	*0,002*
**WSLO_125Y**	Slovenia	Wolf	F	A1	C1	N1	W3	**WH3**	*0,996*	*0,002*	*0,002*
**WSLO_263Y**	Slovenia	Wolf	M	A3	C3	N4	W17	**WH20**	*0,981*	*0,018*	*0,001*
**WSLO_343Y**	Slovenia	Wolf	M	A1	C1	N1	W3	**WH3**	*0,995*	*0,003*	*0,002*
**WSLO_399Y**	Slovenia	Wolf	F	A1	C1	N1	W3	**WH3**	*0,983*	*0,015*	*0,002*
**WSLO_530Y**	Slovenia	Wolf	M	A1	C1	N1	W3	**WH3**	*0,997*	*0,001*	*0,002*
**WSLO_532Y**	Slovenia	Wolf	M	A1	C1	N1	W3	**WH3**	*0,991*	*0,005*	*0,004*
**WSLO_54E**	Slovenia	Wolf	M	A1	C1	N1	W3	**WH3**	*0,996*	*0,003*	*0,001*
**WSLO_6Lu**	Slovenia	Wolf	M	A1	C1	N1	W3	**WH3**	*0,993*	*0,004*	*0,003*
**WSLO_74F**	Slovenia	Wolf	M	A1	C1	N1	W3	**WH3**	*0,996*	*0,002*	*0,002*
**WSLO_AP08CP**	Slovenia	Wolf	M	A3	C3	N4	W17	**WH20**	*0,988*	*0,01*	*0,002*
**WSLO_AP08E1**	Slovenia	Wolf	M	A1	C1	N1	W3	**WH3**	*0,977*	*0,02*	*0,003*
**WSLO_AP08E3**	Slovenia	Wolf	M	A1	C1	N1	W3	**WH3**	*0,996*	*0,003*	*0,001*
**WSLO_AP08E6**	Slovenia	Wolf	M	A1	C1	N1	W3	**WH3**	*0,992*	*0,006*	*0,002*
**WSLO_AP08E8**	Slovenia	Wolf	M	A1	C1	N1	W3	**WH3**	*0,996*	*0,003*	*0,001*
**WSLO_AP08EK**	Slovenia	Wolf	M	A3	C3	N4	W17	**WH20**	*0,996*	*0,002*	*0,002*
**WSLO_EAM**	Slovenia	Wolf	F	A1	C1	N1	W3	**WH3**	*0,995*	*0,003*	*0,002*
**WHR109**	Croatia	Wolf	M						*0,991*	*0,009*	*0,001*
**WHR112**	Croatia	Wolf	F						*0,995*	*0,003*	*0,002*
**WHR115**	Croatia	Wolf	F						*0,991*	*0,003*	*0,006*
**WHR142**	Croatia	Wolf	F						*0,994*	*0,005*	*0,001*
**WHR146**	Croatia	Wolf	M						*0,996*	*0,003*	*0,001*
**WHR165**	Croatia	Wolf	M						*0,996*	*0,002*	*0,002*
**WHR10**	Croatia	Wolf	M						*0,984*	*0,013*	*0,003*
**WHR33**	Croatia	Wolf	M						*0,994*	*0,002*	*0,004*
**WHR48**	Croatia	Wolf	M						*0,782*	*0,005*	*0,213*
**WHR87**	Croatia	Wolf	M						*0,995*	*0,003*	*0,002*
**WHR91**	Croatia	Wolf	M						*0,996*	*0,003*	*0,002*
**WHR96**	Croatia	Wolf	F						*0,995*	*0,003*	*0,002*

**WHRN18**	Croatia	Wolf	M						*0,996*	*0,003*	*0,001*
**WHR137**	Croatia	Wolf	F						*0,996*	*0,002*	*0,002*
**WHR151**	Croatia	Wolf	F						*0,985*	*0,006*	*0,009*
**WHRBH2**	Croatia	Wolf	M						*0,004*	*0,005*	*0,002*
**WHRLI01**	Croatia	Wolf	M						*0,995*	*0,003*	*0,002*
**WHRLI02**	Croatia	Wolf	M						*0,988*	*0,008*	*0,004*
**WHRLI03**	Croatia	Wolf	M						*0,997*	*0,002*	*0,001*
**WHRLI04**	Croatia	Wolf	M						*0,997*	*0,002*	*0,001*
**W184**	Greece	Wolf	M	A2	C1	N2	W5	**WH6**	*0,997*	*0,002*	*0,001*
**W186**	Greece	Wolf	M	A3	C3	N5	W15	**WH17**	*0,985*	*0,013*	*0,002*
**W187**	Greece	Wolf	F	A2	C1	N2	W5	**WH6**	*0,996*	*0,002*	*0,002*
**W322**	Greece	Wolf	F	A1	C2	N2	W4	**WH4**	*0,98*	*0,017*	*0,003*
**W323**	Greece	Wolf	F	A1	C2	N2	W4	**WH4**	*0,987*	*0,01*	*0,003*
**W325**	Greece	Wolf	F	A3	C3	N4	W16	**WH18**	*0,918*	*0,002*	*0,08*
**W326**	Greece	Wolf	M	A3	C3	N5	W15	**WH17**	*0,989*	*0,005*	*0,006*
**W327**	Greece	Wolf		A1	C1	N6	W14	**WH16**			
**W379**	Greece	Wolf		A1	C1	N6	W14	**WH16**			
**W380**	Greece	Wolf	F	A1	C2	N2	W4	**WH4**			
**W381**	Greece	Wolf		A3	C3	N4	W14	**WH15**			
**W382**	Greece	Wolf	F	A2	C1	N2	W5	**WH6**	*0,989*	*0,009*	*0,002*
**W383**	Greece	Wolf	M	A1	C2	N2	W4	**WH4**	*0,987*	*0,01*	*0,003*
**W384**	Greece	Wolf		A2	C1	N2	W5	**WH6**			
**W507**	Greece	Wolf	M	A3	C3	N4	W16	**WH18**	*0,996*	*0,002*	*0,002*
**WBG198**	Bulgaria	Wolf	M	A3	C3	N4	W16	**WH18**	*0,995*	*0,002*	*0,003*
**WBG208**	Bulgaria	Wolf	F	A1	C1	N1	W2	**WH2**	*0,977*	*0,003*	*0,02*
**WBG209**	Bulgaria	Wolf	M	A2	C1	N2	W5	**WH6**	*0,955*	*0,043*	*0,002*
**WBG211**	Bulgaria	Wolf	M	A3	C3	N4	W16	**WH18**	*0,995*	*0,003*	*0,002*
**WBG213**	Bulgaria	Wolf	M	A3	C3	N4	W16	**WH18**	*0,939*	*0,052*	*0,009*
**WBG214**	Bulgaria	Wolf	M	A2	C1	N2	W5	**WH6**	*0,957*	*0,038*	*0,005*
**WBG215**	Bulgaria	Wolf	M	A1	C1	N3	W9	**WH9**	*0,995*	*0,003*	*0,002*
**WBG220**	Bulgaria	Wolf	M	A2	C1	N2	W5	**WH6**	*0,986*	*0,008*	*0,006*
**WBG225**	Bulgaria	Wolf	M	A1	C1	N1	W1	**WH1**	*0,988*	*0,004*	*0,008*
**WBG226**	Bulgaria	Wolf	M	A2	C1	N2	W5	**WH6**	*0,886*	*0,049*	*0,065*
**WBG227**	Bulgaria	Wolf	M	A3	C3	N4	W16	**WH18**	*0,993*	*0,003*	*0,004*
**WBG228**	Bulgaria	Wolf	M	A3	C3	N4	W16	**WH18**	*0,991*	*0,006*	*0,003*
**WBG1502**	Bulgaria	Wolf	M	A1	C1	N3	W9	**WH9**	*0,992*	*0,005*	*0,003*
**WBG1505**	Bulgaria	Wolf	M	A1	C1	N3	W9	**WH9**	*0,975*	*0,023*	*0,002*
**WBG1507**	Bulgaria	Wolf	M	A1	C1	N3	W9	**WH9**	*0,931*	*0,059*	*0,01*
**WBG1510**	Bulgaria	Wolf	F	A1	C1	N3	W9	**WH9**	*0,995*	*0,003*	*0,002*
**WBG1514**	Bulgaria	Wolf	M	A2	C1	N2	W5	**WH6**	*0,955*	*0,041*	*0,004*
**WCZ1350M**	Czech R.	Wolf	M						*0,989*	*0,003*	*0,008*
**WCZ1358F**	Czech R.	Wolf	F						*0,958*	*0,009*	*0,033*
**WCZ1366M**	Czech R.	Wolf	M						*0,99*	*0,004*	*0,006*
**WCZ1374M**	Czech R.	Wolf	M						*0,993*	*0,003*	*0,004*
**WCZ1398M**	Czech R.	Wolf	M						*0,993*	*0,003*	*0,004*
**WCZ1406M**	Czech R.	Wolf	M						*0,994*	*0,002*	*0,004*
**WCZ1414F**	Czech R.	Wolf	F						*0,993*	*0,003*	*0,004*
**253**	Czech R.	Wolf	F						*0,992*	*0,002*	*0,005*
**254**	Czech R.	Wolf	M						*0,993*	*0,004*	*0,003*
**255**	Czech R.	Wolf	M						*0,987*	*0,011*	*0,002*
**608**	Czech R.	Wolf	F						*0,996*	*0,002*	*0,002*
**D349**	Czech R.	Wolf	F						*0,995*	*0,002*	*0,003*
**D351**	Czech R.	Wolf	M						*0,995*	*0,003*	*0,002*

**D352**	Czech R.	Wolf	M							*0,997*	*0,002*	*0,001*
**D355**	Czech R.	Wolf	F							*0,994*	*0,003*	*0,003*
**D389**	Czech R.	Wolf	F							*0,994*	*0,002*	*0,004*
**D390**	Czech R.	Wolf	M							*0,995*	*0,002*	*0,003*
**D454**	Czech R.	Wolf	F							*0,994*	*0,003*	*0,003*
**D591**	Czech R.	Wolf	F							*0,996*	*0,002*	*0,002*
**D592**	Czech R.	Wolf	F							*0,996*	*0,002*	*0,002*
**140**	Poland	Wolf		A1	C1	N6	W7	**WH7**				
**141**	Poland	Wolf		A1	C1	N6	W7	**WH7**				
**158**	Poland	Wolf		A1	C8	N12	W23	**WH27**				
**247**	Poland	Wolf		A1	C1	N6	W7	**WH7**				
**346**	Poland	Wolf		A1	C1	N6	W7	**WH7**				
**379**	Poland	Wolf		A1	C8	N12	W23	**WH27**				
**447**	Poland	Wolf		A1	C1	N6	W7	**WH7**				
**502**	Poland	Wolf		A1	C1	N3	W9	**WH9**				
**585**	Poland	Wolf		A1	C8	N12	W23	**WH27**				
**812**	Poland	Wolf		A1	C1	N3	W9	**WH9**				
**874**	Poland	Wolf		A1	C1	N1	W22	**WH26**				
**962**	Poland	Wolf		A1	C8	N12	W23	**WH27**				
**1179**	Poland	Wolf		A1	C1	N6	W7	**WH7**				
**2223**	Poland	Wolf		A3	C3	N4	W18	**WH21**				
**C95**	Poland	Wolf		A1	C8	N12	W23	**WH27**				
**C104**	Poland	Wolf		A1	C1	N6	W7	**WH7**				
**E516**	Estonia	Wolf	M	A1	C1	N6	W7	**WH7**	*0,994*	*0,002*	*0,004*	
**E530**	Estonia	Wolf	F	A1	C1	N11	W24	**WH28**	*0,995*	*0,002*	*0,003*	
**E533**	Estonia	Wolf	M	A1	C1	N6	W7	**WH7**	*0,948*	*0,013*	*0,038*	
**E535**	Estonia	Wolf	F	A1	C8	N12	W23	**WH27**	*0,984*	*0,011*	*0,005*	
**E560**	Estonia	Wolf	M	A1	C1	N6	W7	**WH7**	*0,995*	*0,003*	*0,002*	
**E568**	Estonia	Hybrid	M	A1	C8	N12	W23	**WH27**	*0,927*	*0,001*	*0,072*	
**E576**	Estonia	Wolf	F	A1	C1	N6	W7	**WH7**	*0,996*	*0,001*	*0,003*	
**E603**	Estonia	Wolf	M	A1	C1	N6	W7	**WH7**	*0,996*	*0,002*	*0,002*	
**E623**	Estonia	Wolf	F	A1	C1	N6	W7	**WH7**	*0,986*	*0,009*	*0,005*	
**E629**	Estonia	Wolf	F	A1	C1	N6	W7	**WH7**	*0,994*	*0,002*	*0,004*	
**L165**	Latvia	Wolf	F	A1	C1	N6	W7	**WH7**	*0,952*	*0,032*	*0,016*	
**L253**	Latvia	Wolf	F	A1	C1	N6	W7	**WH7**	*0,993*	*0,002*	*0,005*	
**L404**	Latvia	Wolf	M	A1	C1	N6	W7	**WH7**	*0,992*	*0,005*	*0,003*	
**L406**	Latvia	Wolf	F	A1	C1	N6	W7	**WH7**	*0,989*	*0,001*	*0,01*	
**L407**	Latvia	Wolf	F	A1	C8	N12	W23	**WH27**	*0,981*	*0,003*	*0,016*	
**L413**	Latvia	Wolf	F	A1	C1	N6	W7	**WH7**	*0,897*	*0,1*	*0,003*	
**L416**	Latvia	Wolf	M	A1	C1	N6	W7	**WH7**	*0,992*	*0,005*	*0,003*	
**L419**	Latvia	Wolf	M	A1	C1	N6	W7	**WH7**	*0,996*	*0,002*	*0,002*	
**L424**	Latvia	Wolf	F	A1	C1	N6	W7	**WH7**	*0,986*	*0,006*	*0,008*	
**L426**	Latvia	Wolf	M	A1	C1	N6	W7	**WH7**	*0,934*	*0,029*	*0,037*	
**W317**	Finland	Wolf	F	A4	C5	N7	W13	**WH12**	*0,989*	*0,007*	*0,004*	
**W318**	Finland	Wolf	M	A4	C5	N7	W13	**WH12**	*0,992*	*0,002*	*0,006*	
**W319**	Finland	Wolf	M	A4	C5	N8	W13	**WH13**	*0,996*	*0,001*	*0,003*	
**W320**	Finland	Wolf	F	A1	C1	N6	W8	**WH8**	*0,985*	*0,008*	*0,007*	
**W321**	Finland	Wolf	F	A1	C1	N6	W7	**WH7**	*0,994*	*0,002*	*0,004*	
**W396**	Finland	Wolf	M	A4	C5	N7	W13	**WH12**	*0,911*	*0,086*	*0,003*	
**W397**	Finland	Wolf	M	A1	C1	N6	W8	**WH8**	*0,995*	*0,003*	*0,002*	
**W398**	Finland	Wolf	F	A1	C1	N6	W8	**WH8**	*0,997*	*0,001*	*0,002*	
**W399**	Finland	Wolf	M	A4	C5	N7	W13	**WH12**	*0,976*	*0,006*	*0,018*	
**D008F**	Italy	Dog	F				D9		*0,002*	*0,001*	*0,997*	

**D100M**	Italy	Dog	M				D5-6		*0,023*	*0,093*	*0,884*
**D104F**	Italy	Dog	F						*0,001*	*0,001*	*0,998*
**D105M**	Italy	Dog	M				D14		*0,002*	*0,002*	*0,996*
**D107M**	Italy	Dog	M				D13-15		*0,063*	*0,001*	*0,936*
**D112M**	Italy	Dog	M				D14		*0,009*	*0,002*	*0,989*
**D115F**	Italy	Dog	F				D14		*0,002*	*0,001*	*0,997*
**D116F**	Italy	Dog	F				D1		*0,009*	*0,003*	*0,988*
**D117F**	Italy	Dog	F				D1		*0,003*	*0,001*	*0,996*
**D121M**	Italy	Dog	M				D8		*0,007*	*0,003*	*0,99*
**D122M**	Italy	Dog	M				D9		*0,007*	*0,001*	*0,992*
**D123M**	Italy	Dog	M				D14		*0,002*	*0,002*	*0,996*
**D124F**	Italy	Dog	F				D10		*0,013*	*0,008*	*0,979*
**D125F**	Italy	Dog	F				D10		*0,013*	*0,003*	*0,984*
**D855M**	Italy	Dog	M				D1		*0,005*	*0,005*	*0,99*
**D857M**	Italy	Dog	M				D13-15		*0,023*	*0,043*	*0,934*
**D859M**	Italy	Dog	M				D1		*0,003*	*0,002*	*0,995*
**D860F**	Italy	Dog	F				D13-15		*0,002*	*0,001*	*0,997*
**D868M**	Italy	Dog	M				D13-15		*0,006*	*0,002*	*0,992*
**D872F**	Italy	Dog	F				D1		*0,002*	*0,001*	*0,997*
**D880M**	Italy	Dog	M						*0,065*	*0,024*	*0,911*
**D887F**	Italy	Dog	F				D8		*0,004*	*0,006*	*0,99*
**D889F**	Italy	Dog	F				D8		*0,003*	*0,003*	*0,994*
**D890M**	Italy	Dog	M				D13-15		*0,013*	*0,012*	*0,974*
**D1290M**	Italy	Dog	M				D13-15		*0,003*	*0,002*	*0,995*
**D1291F**	Italy	Dog	F				D9-D10		*0,003*	*0,003*	*0,994*
**D1292M**	Italy	Dog	M				D13-15		*0,003*	*0,013*	*0,984*
**D1293F**	Italy	Dog	F				D1		*0,021*	*0,032*	*0,947*
**D1294M**	Italy	Dog	M	A14	C1	N20	D2	**DH8**	*0,002*	*0,001*	*0,997*
**D1295F**	Italy	Dog	F				D13-15		*0,004*	*0,002*	*0,994*
**D1296M**	Italy	Dog	M				D13-15		*0,007*	*0,005*	*0,988*
**D1431F**	Italy	Dog	F						*0,008*	*0,006*	*0,986*
**D1432F**	Italy	Dog	F				D1		*0,203*	*0,032*	*0,765*
**D1442F**	Italy	Dog	F				D13		*0,146*	*0,005*	*0,848*
**D1449M**	Italy	Dog	M						*0,009*	*0,049*	*0,941*
**D1450M**	Italy	Dog	M						*0,003*	*0,003*	*0,994*
**D1460M**	Italy	Dog	M				D13		*0,006*	*0,002*	*0,992*
**D1564**	Italy	Dog							*0,006*	*0,004*	*0,99*
**D1578F**	Italy	Dog	F						*0,022*	*0,001*	*0,977*
**D1715M**	Italy	Dog	M						*0,008*	*0,002*	*0,99*
**D1716F**	Italy	Dog	F						*0,003*	*0,004*	*0,993*
**D1819M**	Italy	Dog	M						*0,003*	*0,01*	*0,987*
**D1905F**	Italy	Dog	F						*0,004*	*0,001*	*0,995*
**D1248F**	Italy	Dog	F						*0,002*	*0,001*	*0,997*
**D1249M**	Italy	Dog	M				D5-6		*0,002*	*0,002*	*0,996*
**D1250M**	Italy	Dog	M				D1		*0,002*	*0,001*	*0,997*
**D1251F**	Italy	Dog	F	A16	C12	N23	D9	**DH20**	*0,001*	*0,001*	*0,998*
**D1252F**	Italy	Dog	F				D1		*0,002*	*0,001*	*0,997*
**D1253F**	Italy	Dog	F				D14		*0,003*	*0,002*	*0,995*
**D1254F**	Italy	Dog	F						*0,006*	*0,002*	*0,992*
**D1255M**	Italy	Dog	M	A16	C12	N23	D9	**DH20**	*0,001*	*0,001*	*0,998*

**D1256F**	Italy	Dog	F						*0,002*	*0,001*	*0,997*
**D1257F**	Italy	Dog	F				D1		*0,003*	*0,002*	*0,995*
**D1258F**	Italy	Dog	F						*0,001*	*0,001*	*0,998*
**D1259F**	Italy	Dog	F				D5-6		*0,001*	*0,001*	*0,998*
**D1260M**	Italy	Dog	M						*0,002*	*0,001*	*0,997*
**D1261M**	Italy	Dog	M				D13-15		*0,004*	*0,002*	*0,994*
**D1262M**	Italy	Dog	M	A14	C1	N20	D2	**DH8**	*0,002*	*0,001*	*0,997*
**D1263F**	Italy	Dog	F						*0,002*	*0,001*	*0,997*
**D1264F**	Italy	Dog	F				D13-15		*0,019*	*0,002*	*0,979*
**D1265M**	Italy	Dog	M	A16	C12	N23	D9	**DH20**	*0,001*	*0,001*	*0,998*
**D1266M**	Italy	Dog	M	A14	C1	N20	D2	**DH8**	*0,002*	*0,002*	*0,996*
**D1267M**	Italy	Dog	M						*0,002*	*0,001*	*0,997*
**D1268M**	Italy	Dog	M						*0,004*	*0,002*	*0,994*
**D1269F**	Italy	Dog	F				D9-D10		*0,001*	*0,001*	*0,998*
**D1270M**	Italy	Dog	M						*0,002*	*0,001*	*0,997*
**D1271F**	Italy	Dog	F	A16	C12	N23	D9	**DH20**	*0,001*	*0,001*	*0,998*
**D1272F**	Italy	Dog	F						*0,001*	*0,001*	*0,998*
**D1273F**	Italy	Dog	F	A14	C1	N20	D2	**DH8**	*0,002*	*0,001*	*0,997*
**H183_EST**	Estonia	Hybrid	F	A1	C1	N6	W7	**WH7**	*0,517*	*0,003*	*0,48*
**H185_EST**	Estonia	Hybrid	F	A1	C1	N6	W7	**WH7**	*0,412*	*0,003*	*0,585*
**H186_EST**	Estonia	Hybrid	M	A1	C1	N6	W7	**WH7**	*0,393*	*0,001*	*0,606*
**H255_EST**	Estonia	Hybrid	F	A1	C1	N6	W7	**WH7**	*0,35*	*0,004*	*0,646*
**H256_EST**	Estonia	Hybrid	F	A1	C1	N6	W7	**WH7**	*0,358*	*0,001*	*0,641*
**H257_EST**	Estonia	Hybrid	F	A1	C1	N6	W7	**WH7**	*0,424*	*0,006*	*0,57*
**HHR07M**	Croatia	Hybrid	M				W9		*0,99*	*0,003*	*0,007*
**HHR13M**	Croatia	Hybrid	M				W3		*0,992*	*0,006*	*0,002*
**HHR32M**	Croatia	Hybrid	M				W16		*0,738*	*0,003*	*0,259*
**HHR51M**	Croatia	Hybrid	M				W16		*0,756*	*0,004*	*0,24*
**HHR52M**	Croatia	Hybrid	M				W17		*0,995*	*0,003*	*0,002*
**HHR56F**	Croatia	Hybrid	F				W17		*0,998*	*0,001*	*0,001*
**HHR58F**	Croatia	Hybrid	F				W9		*0,991*	*0,004*	*0,005*
**HHR59F**	Croatia	Hybrid	F				W3		*0,992*	*0,005*	*0,003*
**HHR66F**	Croatia	Hybrid	F				W3		*0,994*	*0,004*	*0,002*
**HHR67M**	Croatia	Hybrid	M				W17		*0,99*	*0,004*	*0,006*
**HHR69F**	Croatia	Hybrid	F				W16		*0,966*	*0,028*	*0,006*
**HHR71M**	Croatia	Hybrid	M				W17		*0,998*	*0,001*	*0,001*
**HHR72M**	Croatia	Hybrid	M				W3		*0,995*	*0,003*	*0,002*
**HHR73M**	Croatia	Hybrid	M				W16		*0,988*	*0,004*	*0,007*
**HHR74F**	Croatia	Hybrid	F				W16		*0,977*	*0,018*	*0,005*
**HHR75F**	Croatia	Hybrid	F				W3		*0,799*	*0,002*	*0,199*
**HHR76M**	Croatia	Hybrid	M				W9		*0,996*	*0,002*	*0,002*
**HHR101M**	Croatia	Hybrid	M				W17		*0,991*	*0,006*	*0,003*
**HHR127F**	Croatia	Hybrid	F				W3		*0,996*	*0,002*	*0,002*
**HHRCAK1F**	Croatia	Hybrid	F						*0,99*	*0,005*	*0,005*
**HHRCG01F**	Croatia	Hybrid	F				W17		*0,985*	*0,012*	*0,003*
**HHR103F**	Croatia	Hybrid	F				W17		*0,992*	*0,003*	*0,005*
**HHR111M**	Croatia	Hybrid	M				W3		*0,993*	*0,004*	*0,003*
**HHR157M**	Croatia	Hybrid	M				W3		*0,991*	*0,002*	*0,007*
**HHR162F**	Croatia	Hybrid	F						*0,995*	*0,001*	*0,004*
**HHR170F**	Croatia	Hybrid	M				W16		*0,994*	*0,004*	*0,002*
**H131M**	Italy	Hybrid	M						*0,002*	*0,489*	*0,51*

**H520M**	Italy	Hybrid	M	A3	C3	N5	W14	**WH14**	*0,007*	*0,651*	*0,342*
**H535M**	Italy	Hybrid	M	A3	C3	N5	W14	**WH14**	*0,008*	*0,92*	*0,071*
**H586F**	Italy	Hybrid	F	A3	C3	N5	W14	**WH14**	*0,001*	*0,998*	*0,001*
**H587F**	Italy	Hybrid	F	A3	C3	N5	W14	**WH14**	*0,005*	*0,776*	*0,218*
**H667M**	Italy	Hybrid	M	A3	C3	N5	W14	**WH14**	*0,001*	*0,998*	*0,001*
**H893F**	Italy	Hybrid	F	A3	C3	N5	W14	**WH14**	*0,001*	*0,998*	*0,001*
**H908M**	Italy	Hybrid	M	A3	C3	N5	W14	**WH14**	*0,002*	*0,997*	*0,001*
**H912M**	Italy	Hybrid	M	A3	C3	N5	W14	**WH14**	*0,001*	*0,997*	*0,002*
**IBRIDO1F**	Italy	Hybrid	F				W14		*0,006*	*0,937*	*0,057*
**H950F**	Italy	Hybrid	F	A3	C3	N5	W14	**WH14**	*0,002*	*0,657*	*0,341*
**H955F**	Italy	Hybrid	F	A3	C3	N5	W14	**WH14**	*0,004*	*0,847*	*0,149*
**WRE10M**	Italy	Hybrid	M				W14		*0,001*	*0,998*	*0,001*
**H1152M**	Italy	Hybrid	M	A3	C3	N5	W14	**WH14**	*0,005*	*0,963*	*0,032*
**H1053M**	Italy	Hybrid	M	A3	C3	N5	W14	**WH14**	*0,002*	*0,996*	*0,002*
**H1153M**	Italy	Hybrid	M				W14		*0,031*	*0,696*	*0,273*
**H1224M**	Italy	Hybrid	M	A3	C3	N5	W14	**WH14**	*0,005*	*0,96*	*0,035*
**WPR52F**	Italy	Hybrid	F	A3	C3	N5	W14	**WH14**	*0,002*	*0,983*	*0,015*
**H1239M**	Italy	Hybrid	M	A3	C3	N5	W14	**WH14**	*0,003*	*0,887*	*0,11*
**H1433M**	Italy	Hybrid	M	A3	C3	N5	W14	**WH14**	*0,005*	*0,89*	*0,104*
**H1440F**	Italy	Hybrid	F	A3	C3	N5	W14	**WH14**	*0,002*	*0,991*	*0,007*
**H1441F**	Italy	Hybrid	F	A3	C3	N5	W14	**WH14**	*0,003*	*0,99*	*0,007*
**H1443F**	Italy	Hybrid	F	A3	C3	N5	W14	**WH14**	*0,009*	*0,987*	*0,004*
**H1453M**	Italy	Hybrid	M	A3	C3	N5	W14	**WH14**	*0,002*	*0,997*	*0,001*
**H1456M**	Italy	Hybrid	M	A3	C3	N5	W14	**WH14**	*0,008*	*0,964*	*0,028*
**H1458M**	Italy	Hybrid	M				W14		*0,035*	*0,815*	*0,15*
**H1468F**	Italy	Hybrid	F	A3	C3	N5	W14	**WH14**	*0,003*	*0,996*	*0,001*
**H1299M**	Italy	Hybrid	M	A3	C3	N5	W14	**WH14**	*0,015*	*0,955*	*0,03*
**H1470M**	Italy	Hybrid	M	A3	C3	N5	W14	**WH14**	*0,002*	*0,995*	*0,003*
**H1471M**	Italy	Hybrid	M	A3	C3	N5	W14	**WH14**	*0,001*	*0,995*	*0,004*
**H1473M**	Italy	Hybrid	M	A3	C3	N5	W14	**WH14**	*0,092*	*0,807*	*0,101*
**H1474F**	Italy	Hybrid	F	A3	C3	N5	W14	**WH14**	*0,002*	*0,994*	*0,004*
**H1475M**	Italy	Hybrid	M	A3	C3	N5	W14	**WH14**	*0,002*	*0,997*	*0,001*
**H1545F**	Italy	Hybrid	F	A3	C3	N5	W14	**WH14**	*0,004*	*0,983*	*0,013*
**H1551M**	Italy	Hybrid	M	A3	C3	N5	W14	**WH14**	*0,001*	*0,998*	*0,001*
**H1569M**	Italy	Hybrid	M	A3	C3	N5	W14	**WH14**	*0,002*	*0,994*	*0,004*
**H1571F**	Italy	Hybrid	F	A3	C3	N5	W14	**WH14**	*0,035*	*0,955*	*0,01*
**H1572M**	Italy	Hybrid	M				W14		*0,103*	*0,893*	*0,004*
**H1589M**	Italy	Hybrid	M	A3	C3	N5	W14	**WH14**	*0,002*	*0,993*	*0,005*
**H1593M**	Italy	Hybrid	M				W14		*0,004*	*0,993*	*0,003*
**H1602M**	Italy	Hybrid	M	A3	C3	N5	W14	**WH14**	*0,009*	*0,978*	*0,013*
**H1640F**	Italy	Hybrid	F	A3	C3	N5	W14	**WH14**	*0,003*	*0,839*	*0,158*
**H1656F**	Italy	Hybrid	F	A3	C3	N5	W14	**WH14**	*0,007*	*0,989*	*0,004*
**H1657M**	Italy	Hybrid	M	A3	C3	N5	W14	**WH14**	*0,002*	*0,997*	*0,001*
**H1660F**	Italy	Hybrid	F	A3	C3	N5	W14	**WH14**	*0,002*	*0,972*	*0,026*
**H1664F**	Italy	Hybrid	F				W14		*0,002*	*0,993*	*0,005*
**H1670M**	Italy	Hybrid	M	A3	C3	N5	W14	**WH14**	*0,002*	*0,997*	*0,001*
**H1674M**	Italy	Hybrid	M	A3	C3	N5	W14	**WH14**	*0,001*	*0,998*	*0,001*
**H1675F**	Italy	Hybrid	F				W14		*0,001*	*0,998*	*0,001*
**H1676M**	Italy	Hybrid	M				W14		*0,001*	*0,998*	*0,001*
**H1677M**	Italy	Hybrid	M				W14		*0,001*	*0,998*	*0,001*
**H1678F**	Italy	Hybrid	F				W14		*0,001*	*0,998*	*0,001*
**H1679M**	Italy	Hybrid	M				W14		*0,001*	*0,998*	*0,001*
**H1680F**	Italy	Hybrid	F				W14		*0,001*	*0,998*	*0,001*

**H1681M**	Italy	Hybrid	M	W14	*0,001*	*0,998*	*0,001*
**H1682M**	Italy	Hybrid	M	W14	*0,001*	*0,998*	*0,001*
**H1683F**	Italy	Hybrid	F	W14	*0,001*	*0,998*	*0,001*
**H1718M**	Italy	Hybrid	M	W14	*0,003*	*0,89*	*0,107*
**H1725M**	Italy	Hybrid	M		*0,001*	*0,998*	*0,001*
**H1726M**	Italy	Hybrid	M	W14	*0,003*	*0,993*	*0,004*
**H1728M**	Italy	Hybrid	M	W14	*0,013*	*0,98*	*0,007*
**H1817F**	Italy	Hybrid	F	W14	*0,096*	*0,849*	*0,056*
**H1864M**	Italy	Hybrid	M	W14	*0,005*	*0,994*	*0,001*
**H865M**	Italy	Hybrid	M	W14	*0,004*	*0,989*	*0,007*
**H1869M**	Italy	Hybrid	M	W14	*0,009*	*0,919*	*0,072*
**H1895M**	Italy	Hybrid	M	W14	*0,001*	*0,441*	*0,558*
**H1896F**	Italy	Hybrid	F	W14	*0,002*	*0,456*	*0,542*
**H1917F**	Italy	Hybrid	F	W14	*0,014*	*0,7*	*0,287*
