# Supplementary material for: Combining phylogenetic and demographic inferences to assess the origin of the genetic diversity in an isolated wolf population
Source: PLoS One. 2017 May 10;12(5):e0176560. doi: 10.1371/journal.pone.0176560 (PMC5425034; doi:10.1371/journal.pone.0176560)
Supplement: S3 Table — For every sample is shown: the accession number, country of origin of the sequenced sample (if available), taxon, dog breed (if available), and clade of memberships (BEAST analysis), and haplotypes at different genic regions. (PDF) [file pone.0176560.s010.pdf]

S3 Table. List of mtDNA sequences downloaded from the GenBank showing: the accession number, country of origin of the sequenced sample (if available), taxon, dog breed (if available), and clade of memberships (BEAST analysis), and haplotypes at different genic regions.

Acc.#	Country	Taxon	Breed	Dog clade	ATP6	COIII	ND4	CR	MF
KF661041	China	Wolf	---	---	A1	C1	N2	W26	WH31
KF661043	India	Wolf	---	---	A6	C1	N2	W29	WH34
KF661047	Ukraine	Wolf	---	---	A1	C1	N2	W4	WH5
KF661050	Oman	Wolf	---	---	A7	C7	N14	W38	WH44
KF661051	Iran	Wolf	---	---	A7	C7	N14	W28	WH33
KF661052	Sweden	Wolf	---	---	A9	C1	N6	W27	WH32
KF661054	Croatia	Wolf	---	---	A1	C1	N2	W10	WH10
KF661055	Israel	Wolf	---	---	A1	C1	N2	W11	WH11
KF661056	Canada	Wolf	---	---	A10	C1	N16	W30	WH35
KF661058	Alaska	Wolf	---	---	A1	C1	N15	W34	WH40
KF661060	Mexico	Wolf	---	---	A11	C6	N10	W36	WH42
KF661062	Canada	Wolf	---	---	A10	C1	N17	W30	WH36
KF661064	USA	Wolf	---	---	A5	C1	N2	W35	WH41
KF661065	Mexico	Wolf	---	---	A11	C6	N10	W37	WH43
KF661066	Alaska	Wolf	---	---	A5	C1	N2	W32	WH38
KF661071	Alaska	Wolf	---	---	A5	C1	N2	W33	WH39
KF661074	Canada	Wolf	---	---	A5	C1	N2	W31	WH37
DQ480506	Saudi Arabia	Wolf	---	---	A8	C5	N13	W25	WH30
KF661080	Belgium	Ancient	---	---	B30k	B30k	B30k	B30k	B30k
KF661081	Russia	Ancient	---	---	R18k	R18k	R18k	R18k	R18k
KF661085	Russia	Ancient	---	---	R22k	R22k	R22k	R22k	R22k
KF661087	Switzerland	Ancient	---	---	S14.5k	S14.5k	N4	S14.5k	S14.5k
AB499816	Japan	Dog	Kishu	2	A38	C18	N42	D65	DH65
AB499817	Japan	Dog	Husky	2	A39	C19	N43	D66	DH66
AY656737	North America	Dog	Basenji	2	A12	C12	N18	Basenji	Basenji
AY656738	North America	Dog	Jack Russel Terrier	2	A12	C12	N19	D5-D6	DH9
AY656739	North America	Dog	Poodle	4	A14	C1	N20	D1	DH1
AY656740	North America	Dog	Kerry Blue Terrier	4	A14	C1	N20	D1	DH1
AY656741	North America	Dog	Irish Setter	2	A16	C12	N18	D9	DH19
AY656742	North America	Dog	Old English Sheepdog	2	A27	C12	N18	D67	DH67
AY656743	North America	Dog	Saint Bernard	4	A14	C1	N27	D1	DH2
AY656744	North America	Dog	English Springer Spaniel	2	A13	C12	N18	D13	DH23
AY656745	North America	Dog	English Springer Spaniel	4	A14	C1	N20	D68	DH68
AY656746	North America	Dog	Standard Schnauzer	2	A12	C12	N32	D8	DH15
AY656747	North America	Dog	Welsh Springer Spaniel	2	A13	C12	N18	D53	DH34
AY656748	North America	Dog	Airdale Terrier	2	A12	C12	N19	D5-D6	DH9
AY656749	North America	Dog	Saint Bernard	2	A16	C12	N18	D9	DH19
AY656750	North America	Dog	Leonberger	2	A12	C12	N32	D8	DH15
AY656751	North America	Dog	Gordon Setter	2	A12	C12	N30	D5-D6	DH10
AY656752	North America	Dog	Standard Schnauzer	4	A14	C1	N20	D69	DH69
AY656753	North America	Dog	Irish Setter	2	A23	C12	N18	D13	DH24
AY656754	North America	Dog	Chinese Crested	2	A13	C12	N18	D70	DH70
AY656755	North America	Dog	Sapsaree	2	A13	C13	N18	D14	DH29
CFU96639	North America	Dog	NA	2	A26	C13	N18	D14	DH30
DQ480489	Sweden	Dog	German Shepherd	3	A11	C5	N25	D54	DH38
DQ480490	Sweden	Dog	Flat Coated Retriever	4	A14	C1	N20	D55	DH39
DQ480491	Sweden	Dog	Irish Setter	2	A16	C11	N18	D9	DH18
DQ480492	Sweden	Dog	Jamthund	3	A18	C9	N24	D52	DH33
DQ480493	Sweden	Dog	Black Russian Terrier	3	A11	C5	N26	D56	DH40
DQ480494	Sweden	Dog	Poodle	4	A14	C1	N20	D1	DH1
DQ480495	Sweden	Dog	Cocker Spaniel	2	A13	C12	N18	D53	DH34
DQ480496	Sweden	Dog	Irish Soft Coated Wheaten Terrier	2	A13	C12	N18	D11	DH22
DQ480497	Sweden	Dog	West Highland White Terrier	2	A17	C12	N22	D8	DH14
DQ480498	Sweden	Dog	Miniature Schnauzer	2	A12	C12	N19	D5-D6	DH9
DQ480499	Sweden	Dog	Siberian Husky	2	A15	C10	N21	D10	DH21
DQ480500	Sweden	Dog	Shetland Sheepdog	4	A14	C1	N20	D1	DH1
DQ480501	Sweden	Dog	Jamthund	3	A11	C5	N26	D56	DH40
DQ480502	Sweden	Dog	Jamthund	1	A18	C9	N24	D51	DH32
EU408245	North America	Dog	Akita	2	A12	C15	N30	D57	DH46
EU408246	North America	Dog	American Cocker Spaniel	2	A13	C12	N18	D53	DH34

EU408247	North America	Dog	Australian Terrier	4	A19	C1	N20	D1	DH3
EU408248	North America	Dog	Australian Shepherd	2	A13	C12	N18	D14	DH31
EU408249	North America	Dog	Australian Shepherd	2	A23	C12	N18	D13	DH24
EU408250	North America	Dog	Bichon Frise	2	A12	C12	N19	D5-D6	DH9
EU408251	North America	Dog	Blue Heeler	3	A11	C5	N36	D56	DH41
EU408252	North America	Dog	Bolognese	4	A14	C1	N20	D1	DH1
EU408253	North America	Dog	Boxer	2	A12	C12	N19	D5-D6	DH9
EU408254	North America	Dog	Basset Hound	4	A14	C1	N20	D1	DH1
EU408255	North America	Dog	Basset Hound	4	A14	C1	N20	D1	DH1
EU408256	North America	Dog	Basset Hound	4	A14	C1	N20	D1	DH1
EU408257	North America	Dog	Brittany Spaniel	2	A12	C12	N19	D5-D6	DH9
EU408258	North America	Dog	Cockapoo	4	A14	C1	N27	D71	DH71
EU408259	North America	Dog	Cockapoo	2	A13	C12	N18	D53	DH34
EU408260	North America	Dog	Cardigan Corgi	4	A14	C1	N20	D1	DH1
EU408261	North America	Dog	Chihuahua	2	A13	C12	N18	D53	DH34
EU408262	North America	Dog	Chihuahua	2	A40	C10	N44	D72	DH72
EU408263	North America	Dog	Cavalier King Charles Spaniel	2	A12	C12	N19	D5-D6	DH9
EU408264	North America	Dog	Cairn Terrier	2	A13	C12	N18	D11	DH22
EU408265	North America	Dog	Corgi	2	A12	C12	N30	D57	DH47
EU408266	North America	Dog	Cocker Spaniel	2	A23	C12	N18	D13	DH24
EU408267	North America	Dog	Cocker Spaniel	3	A11	C5	N26	D56	DH40
EU408268	North America	Dog	Cocker Spaniel	4	A14	C1	N20	D73	DH73
EU408269	North America	Dog	Doberman Pinscher	4	A14	C1	N20	D1	DH1
EU408270	North America	Dog	Dachshund	2	A13	C12	N18	D14	DH31
EU408271	North America	Dog	Doguede Bordeaux	2	A12	C12	N19	D5-D6	DH9
EU408272	North America	Dog	Dachshund	2	A13	C12	N18	D53	DH34
EU408273	North America	Dog	English Shepherd	2	A13	C12	N18	D53	DH34
EU408274	North America	Dog	English Mastiff	2	A12	C12	N19	D5-D6	DH9
EU408275	North America	Dog	French Bull Dog	2	A12	C12	N33	D8	DH16
EU408276	North America	Dog	Great Dane	2	A12	C12	N32	D8	DH15
EU408277	North America	Dog	German Shepherd	2	A13	C12	N18	D14	DH31
EU408278	North America	Dog	Great Pyrenees	4	A14	C1	N20	D1	DH1
EU408279	North America	Dog	Havanese	3	A29	C5	N26	D56	DH42
EU408280	North America	Dog	Italian Greyhound	2	A12	C12	N19	D5-D6	DH9
EU408281	North America	Dog	Jack Russel	2	A13	C12	N18	D53	DH34
EU408282	North America	Dog	Keenshond	2	A13	C12	N18	D58	DH48
EU408283	North America	Dog	Keenshond	2	A13	C12	N18	D58	DH48
EU408284	North America	Dog	Keenshond	2	A13	C12	N18	D58	DH48
EU408285	North America	Dog	Labradoodle	4	A14	C1	N20	D1	DH1
EU408286	North America	Dog	Miniature Dachshund	2	A23	C12	N18	D13	DH24
EU408287	North America	Dog	Newfoundland	2	A13	C12	N18	D11	DH22
EU408288	North America	Dog	Norwegian Elkhound	1	A32	C9	N24	D59	DH50
EU408289	North America	Dog	Neapolitan Mastiff	2	A16	C12	N18	D9	DH19
EU408290	North America	Dog	Neapolitan Mastiff	2	A16	C12	N18	D9	DH19
EU408291	North America	Dog	Pomeranian	3	A11	C5	N37	D56	DH43
EU408292	North America	Dog	Poodle	4	A14	C1	N20	D1	DH1
EU408293	North America	Dog	PitBull Terrier	3	A11	C5	N39	D60	DH52
EU408294	North America	Dog	Pug	2	A12	C12	N19	D5-D6	DH9
EU408295	North America	Dog	Rottweiler	2	A23	C12	N18	D13	DH24
EU408296	North America	Dog	Rottweiler	2	A23	C12	N18	D13	DH24
EU408297	North America	Dog	Schipperke	4	A14	C1	N20	D55	DH39
EU408298	North America	Dog	Sheltie	2	A13	C12	N18	D53	DH34
EU408299	North America	Dog	Schnauzer	2	A12	C12	N32	D8	DH15
EU408300	North America	Dog	Tibetan Mastiff	2	A41	C20	N18	D74	DH74
EU408301	North America	Dog	Tibetan Spaniel	4	A14	C1	N20	D1	DH1
EU408302	North America	Dog	Toy Poodle	2	A13	C14	N18	D53	DH35
EU408303	North America	Dog	NA	4	A14	C1	N20	D75	DH75
EU408304	North America	Dog	NA	2	A12	C12	N19	D5-D6	DH9
EU408305	North America	Dog	Viszla	2	A27	C12	N18	D53	DH36
EU408306	North America	Dog	West Highland Terrier	2	A13	C12	N18	D11	DH22
EU408307	North America	Dog	Walker Hound	4	A14	C1	N20	D55	DH39
EU408308	North America	Dog	Yorkie Chihuahua	4	A14	C1	N27	D1	DH2
FJ817358	Sweden	Dog	Golden Retriever	2	A12	C12	N30	D5-D6	DH10
FJ817362	Sweden	Dog	Golden Retriever	2	A12	C12	N30	D5-D6	DH10
FJ817363	Sweden	Dog	Golden Retriever	4	A14	C1	N20	D1	DH1
FJ817364	Sweden	Dog	Golden Retriever	4	A14	C1	N20	D76	DH76
JF342807	North America	Dog	---	2	A13	C12	N18	D58	DH48
JF342808	North America	Dog	---	2	A23	C12	N18	D13	DH24
JF342809	North America	Dog	---	4	A14	C1	N20	D1	DH1
JF342810	North America	Dog	---	2	A13	C12	N18	D77	DH77
JF342812	North America	Dog	---	2	A13	C12	N18	D53	DH34
JF342813	North America	Dog	---	2	A13	C14	N18	D53	DH35
JF342814	North America	Dog	---	4	A14	C1	N34	D13	DH25
JF342815	North America	Dog	---	4	A14	C1	N27	D78	DH78
JF342816	North America	Dog	---	2	A34	C16	N18	D61	DH55
JF342817	North America	Dog	---	2	A27	C12	N18	D79	DH79
JF342818	North America	Dog	---	3	A11	C5	N45	D80	DH80

JF342819	North America	Dog	---	2	A12	C12	N19	D5-D6	DH9
JF342820	North America	Dog	---	2	A23	C12	N18	D13	DH24
JF342821	North America	Dog	---	3	A33	C5	N40	D60	DH53
JF342822	North America	Dog	---	2	A13	C12	N18	D81	DH81
JF342823	North America	Dog	---	2	A12	C21	N18	D82	DH82
JF342824	North America	Dog	---	1	A18	C9	N24	D59	DH51
JF342825	North America	Dog	---	4	A14	C1	N20	D1	DH1
JF342826	North America	Dog	---	4	A14	C1	N20	D83	DH83
JF342827	North America	Dog	---	2	A23	C12	N18	D13	DH24
JF342828	North America	Dog	---	2	A13	C12	N18	D53	DH34
JF342829	North America	Dog	---	2	A22	C12	N19	D5-D6	DH11
JF342831	North America	Dog	---	2	A13	C12	N18	D14	DH31
JF342832	North America	Dog	---	2	A12	C12	N19	D5-D6	DH9
JF342833	North America	Dog	---	2	A24	C12	N18	D13	DH26
JF342834	North America	Dog	---	2	A28	C12	N18	D53	DH37
JF342835	North America	Dog	---	2	A23	C12	N18	D13	DH24
JF342836	North America	Dog	---	2	A37	C16	N46	D84	DH84
JF342837	North America	Dog	---	4	A14	C1	N20	D1	DH1
JF342838	North America	Dog	---	---	A1	C1	N2	D62	DH58
JF342839	North America	Dog	---	3	A11	C17	N39	D63	DH60
JF342840	North America	Dog	---	3	A11	C5	N38	D56	DH44
JF342842	North America	Dog	---	4	A14	C1	N20	D1	DH1
JF342843	North America	Dog	---	2	A13	C12	N18	D53	DH34
JF342844	North America	Dog	---	4	A14	C1	N20	D1	DH1
JF342846	North America	Dog	---	2	A27	C12	N18	D53	DH36
JF342847	North America	Dog	---	2	A12	C12	N19	D5-D6	DH9
JF342848	North America	Dog	---	2	A40	C10	N44	D72	DH72
JF342849	North America	Dog	---	4	A14	C1	N20	D1	DH1
JF342850	North America	Dog	---	2	A13	C12	N18	D53	DH34
JF342851	North America	Dog	---	2	A12	C12	N19	D5-D6	DH9
JF342852	North America	Dog	---	2	A42	C10	N47	D85	DH85
JF342853	North America	Dog	---	3	A13	C5	N38	D64	DH62
JF342854	North America	Dog	---	4	A19	C1	N20	D1	DH3
JF342855	North America	Dog	---	2	A16	C12	N18	D9	DH19
JF342856	North America	Dog	---	4	A14	C1	N20	D1	DH1
JF342857	North America	Dog	---	2	A23	C12	N18	D13	DH24
JF342858	North America	Dog	---	2	A23	C12	N18	D13	DH24
JF342859	North America	Dog	---	1	A43	C9	N48	D86	DH86
JF342860	North America	Dog	---	4	A14	C1	N27	D1	DH2
JF342861	North America	Dog	---	4	A14	C1	N20	D1	DH1
JF342862	North America	Dog	---	2	A15	C10	N21	D87	DH87
JF342863	North America	Dog	---	2	A12	C21	N18	D82	DH82
JF342864	North America	Dog	---	4	A14	C1	N20	D88	DH88
JF342865	North America	Dog	---	2	A23	C12	N18	D13	DH24
JF342867	North America	Dog	---	2	A12	C12	N32	D8	DH15
JF342868	North America	Dog	---	3	A11	C5	N49	D89	DH89
JF342869	North America	Dog	---	4	A14	C1	N28	D1	DH4
JF342870	North America	Dog	---	2	A12	C12	N32	D8	DH15
JF342871	North America	Dog	---	2	A12	C12	N32	D8	DH15
JF342872	North America	Dog	---	2	A13	C12	N18	D14	DH31
JF342873	North America	Dog	---	2	A20	C1	N20	D1	DH5
JF342874	North America	Dog	---	4	A27	C12	N18	D53	DH36
JF342875	North America	Dog	---	2	A12	C12	N19	D5-D6	DH9
JF342876	North America	Dog	---	2	A12	C12	N32	D8	DH15
JF342877	North America	Dog	---	2	A23	C12	N18	D13	DH24
JF342878	North America	Dog	---	2	A12	C12	N32	D8	DH15
JF342879	North America	Dog	---	2	A12	C12	N19	D5-D6	DH9
JF342880	North America	Dog	---	3	A11	C5	N26	D64	DH63
JF342881	North America	Dog	---	2	A13	C12	N18	D53	DH34
JF342882	North America	Dog	---	2	A13	C12	N18	D13	DH23
JF342883	North America	Dog	---	2	A13	C14	N18	D53	DH35
JF342884	North America	Dog	---	4	A14	C1	N27	D1	DH2
JF342885	North America	Dog	---	2	A16	C12	N18	D9	DH19
JF342886	North America	Dog	---	2	A13	C12	N18	D13	DH23
JF342887	North America	Dog	---	3	A30	C5	N38	D64	DH64
JF342888	North America	Dog	---	4	A14	C1	N27	D1	DH2
JF342889	North America	Dog	---	2	A13	C14	N18	D53	DH35
JF342890	North America	Dog	---	4	A14	C1	N29	D1	DH6
JF342891	North America	Dog	---	2	A13	C12	N18	D13	DH23
JF342892	North America	Dog	---	3	A35	C16	N41	D61	DH56
JF342893	North America	Dog	---	2	A13	C12	N18	D13	DH23
JF342894	North America	Dog	---	2	A23	C12	N35	D13	DH27
JF342895	North America	Dog	---	2	A12	C12	N32	D8	DH15
JF342896	North America	Dog	---	2	A16	C12	N18	D9	DH19
JF342897	North America	Dog	---	2	A13	C12	N18	D53	DH34
JF342898	North America	Dog	---	4	A14	C1	N27	D1	DH2
JF342899	North America	Dog	---	3	A35	C16	N41	D61	DH56

JF342900	North America	Dog	---	2	A27	C12	N18	D53	DH36
JF342901	North America	Dog	---	2	A12	C12	N19	D5-D6	DH9
JF342902	North America	Dog	---	2	A12	C12	N19	D5-D6	DH9
JF342903	North America	Dog	---	2	A12	C12	N33	D90	DH90
JF342904	North America	Dog	---	2	A12	C12	N19	D5-D6	DH9
JF342905	North America	Dog	---	2	A13	C12	N18	D53	DH34
JF342906	North America	Dog	---	2	A13	C12	N18	D11	DH22
KF907307	North America	Dog	German Shepherd	4	A14	C1	N20	D73	DH73
KF907309	North America	Dog	German Shepherd	3	A11	C5	N39	D63	DH61
KF926378	North America	Dog	German Shepherd	4	A14	C1	N20	D73	DH73
KJ637028	---	Dog	---	2	A13	C13	N18	D14	DH29
KJ637029	---	Dog	---	2	A13	C13	N18	D14	DH29
KJ637030	---	Dog	---	2	A13	C12	N18	D14	DH31
KJ637031	---	Dog	---	2	A13	C12	N18	D14	DH31
KJ637032	---	Dog	---	2	A13	C13	N18	D14	DH29
KJ637033	---	Dog	---	2	A13	C12	N18	D53	DH34
KJ637034	---	Dog	---	2	A13	C12	N18	D53	DH34
KJ637035	---	Dog	---	2	A13	C12	N18	D53	DH34
KJ637036	---	Dog	---	2	A13	C12	N18	D53	DH34
KJ637037	---	Dog	---	2	A13	C12	N18	D53	DH34
KJ637038	---	Dog	---	2	A13	C12	N18	D53	DH34
KJ637039	---	Dog	---	2	A13	C12	N18	D53	DH34
KJ637040	---	Dog	---	2	A13	C12	N18	D53	DH34
KJ637041	---	Dog	---	2	A13	C12	N18	D91	DH91
KJ637042	---	Dog	---	2	A13	C12	N18	D91	DH91
KJ637043	---	Dog	---	2	A13	C12	N18	D92	DH92
KJ637044	---	Dog	---	2	A31	C12	N18	D58	DH49
KJ637045	---	Dog	---	2	A31	C12	N18	D58	DH49
KJ637046	---	Dog	---	2	A13	C12	N18	D53	DH34
KJ637047	---	Dog	---	2	A13	C12	N18	D53	DH34
KJ637048	---	Dog	---	2	A13	C12	N18	D93	DH93
KJ637049	---	Dog	---	2	A13	C12	N18	D53	DH34
KJ637050	---	Dog	---	2	A27	C12	N18	D67	DH67
KJ637051	---	Dog	---	2	A16	C12	N18	D9	DH19
KJ637052	---	Dog	---	2	A16	C12	N18	D9	DH19
KJ637053	---	Dog	---	2	A13	C12	N18	D13	DH23
KJ637054	---	Dog	---	2	A23	C12	N18	D13	DH24
KJ637055	---	Dog	---	2	A23	C12	N18	D13	DH24
KJ637056	---	Dog	---	2	A23	C12	N18	D13	DH24
KJ637057	---	Dog	---	2	A23	C12	N18	D13	DH24
KJ637058	---	Dog	---	2	A23	C12	N18	D13	DH24
KJ637059	---	Dog	---	2	A23	C12	N18	D13	DH24
KJ637060	---	Dog	---	2	A23	C12	N18	D13	DH24
KJ637061	---	Dog	---	2	A13	C12	N18	D13	DH23
KJ637062	---	Dog	---	2	A13	C12	N18	D13	DH23
KJ637063	---	Dog	---	2	A23	C12	N18	D13	DH24
KJ637064	---	Dog	---	2	A23	C12	N18	D13	DH24
KJ637065	---	Dog	---	2	A23	C12	N18	D13	DH24
KJ637066	---	Dog	---	2	A23	C12	N18	D13	DH24
KJ637067	---	Dog	---	2	A25	C12	N18	D13	DH28
KJ637068	---	Dog	---	2	A23	C12	N18	D13	DH24
KJ637069	---	Dog	---	2	A24	C12	N18	D94	DH94
KJ637070	---	Dog	---	2	A23	C12	N18	D13	DH24
KJ637071	---	Dog	---	2	A12	C12	N19	D5-D6	DH9
KJ637072	---	Dog	---	2	A12	C12	N19	D5-D6	DH9
KJ637073	---	Dog	---	2	A12	C12	N19	D5-D6	DH9
KJ637074	---	Dog	---	2	A12	C12	N19	D5-D6	DH9
KJ637075	---	Dog	---	2	A12	C12	N19	D5-D6	DH9
KJ637076	---	Dog	---	2	A12	C12	N19	D5-D6	DH9
KJ637077	---	Dog	---	2	A12	C12	N19	D5-D6	DH9
KJ637078	---	Dog	---	2	A12	C12	N19	D5-D6	DH9
KJ637079	---	Dog	---	2	A12	C12	N19	D5-D6	DH9
KJ637080	---	Dog	---	2	A12	C12	N19	D5-D6	DH9
KJ637081	---	Dog	---	2	A12	C12	N19	D5-D6	DH9
KJ637082	---	Dog	---	2	A12	C12	N19	D5-D6	DH9
KJ637083	---	Dog	---	2	A12	C12	N19	D5-D6	DH9
KJ637084	---	Dog	---	2	A12	C12	N19	D5-D6	DH9
KJ637085	---	Dog	---	2	A12	C12	N19	D5-D6	DH9
KJ637086	---	Dog	---	2	A12	C12	N19	D5-D6	DH9
KJ637087	---	Dog	---	2	A12	C12	N19	D95	DH95
KJ637088	---	Dog	---	2	A12	C12	N19	D5-D6	DH9
KJ637089	---	Dog	---	2	A12	C12	N19	D5-D6	DH9
KJ637090	---	Dog	---	2	A12	C12	N31	D5-D6	DH12
KJ637091	---	Dog	---	2	A12	C12	N19	D5-D6	DH9
KJ637092	---	Dog	---	2	A12	C12	N19	D5-D6	DH9
KJ637093	---	Dog	---	2	A12	C12	N19	D5-D6	DH9
KJ637094	---	Dog	---	2	A12	C12	N19	D5-D6	DH9

KJ637095	---	Dog	---	2	A12	C12	N31	D5-D6	DH12
KJ637096	---	Dog	---	2	A12	C12	N31	D5-D6	DH12
KJ637097	---	Dog	---	2	A12	C12	N30	D5-D6	DH10
KJ637098	---	Dog	---	2	A37	C16	N41	D61	DH57
KJ637099	---	Dog	---	2	A37	C16	N41	D61	DH57
KJ637100	---	Dog	---	2	A12	C12	N32	D8	DH15
KJ637101	---	Dog	---	2	A12	C12	N33	D90	DH90
KJ637102	---	Dog	---	2	A12	C12	N33	D90	DH90
KJ637103	---	Dog	---	2	A17	C12	N50	D8	DH17
KJ637104	---	Dog	---	4	A14	C1	N20	D96	DH96
KJ637105	---	Dog	---	4	A14	C1	N20	D96	DH96
KJ637107	---	Dog	---	4	A14	C22	N20	D97	DH97
KJ637108	---	Dog	---	4	A14	C22	N20	D97	DH97
KJ637109	---	Dog	---	4	A14	C1	N27	D98	DH98
KJ637110	---	Dog	---	4	A14	C1	N20	D62	DH59
KJ637111	---	Dog	---	4	A14	C1	N20	D1	DH1
KJ637112	---	Dog	---	4	A14	C1	N27	D1	DH2
KJ637113	---	Dog	---	4	A14	C1	N20	D1	DH1
KJ637114	---	Dog	---	4	A14	C1	N20	D1	DH1
KJ637115	---	Dog	---	4	A14	C1	N20	D1	DH1
KJ637116	---	Dog	---	4	A21	C1	N20	D1	DH7
KJ637117	---	Dog	---	4	A14	C1	N20	D1	DH1
KJ637118	---	Dog	---	4	A14	C1	N20	D1	DH1
KJ637119	---	Dog	---	4	A14	C1	N20	D1	DH1
KJ637120	---	Dog	---	4	A14	C1	N20	D1	DH1
KJ637121	---	Dog	---	4	A14	C1	N20	D1	DH1
KJ637122	---	Dog	---	4	A14	C1	N20	D1	DH1
KJ637123	---	Dog	---	4	A14	C1	N20	D1	DH1
KJ637124	---	Dog	---	4	A14	C1	N20	D1	DH1
KJ637125	---	Dog	---	4	A14	C1	N20	D1	DH1
KJ637126	---	Dog	---	4	A14	C1	N20	D1	DH1
KJ637127	---	Dog	---	4	A14	C1	N20	D1	DH1
KJ637128	---	Dog	---	4	A14	C1	N20	D1	DH1
KJ637129	---	Dog	---	4	A14	C1	N20	D1	DH1
KJ637130	---	Dog	---	4	A14	C1	N20	D1	DH1
KJ637131	---	Dog	---	4	A14	C1	N20	D1	DH1
KJ637132	---	Dog	---	4	A14	C1	N27	D1	DH2
KJ637133	---	Dog	---	4	A14	C1	N20	D1	DH1
KJ637134	---	Dog	---	4	A14	C1	N20	D88	DH88
KJ637135	---	Dog	---	4	A14	C1	N20	D55	DH39
KJ637136	---	Dog	---	3	A30	C5	N26	D56	DH45
KJ637137	---	Dog	---	3	A11	C5	N26	D56	DH40
KJ637138	---	Dog	---	3	A11	C5	N26	D99	DH99
KJ637139	---	Dog	---	3	A11	C5	N39	D63	DH61
KJ637140	---	Dog	---	3	A11	C5	N39	D63	DH61
KJ637141	---	Dog	---	3	A11	C5	N39	D63	DH61
KJ637142	---	Dog	---	3	A11	C5	N25	D100	DH100
KJ637143	---	Dog	---	3	A11	C5	N25	D100	DH100
KJ637144	---	Dog	---	2	A36	C5	N39	D60	DH54
KJ637145	---	Dog	---	2	A12	C12	N19	D5-D6	DH9
KJ789955	---	Dog	---	2	A13	C12	N51	D101	DH101
KM113774	---	Dog	---	2	A44	C10	N21	D102	DH102
NC_002008	---	Dog	---	2	A26	C13	N18	D14	DH30
DQ480509	USA	Coyote	---		Coyote	Coyote	Coyote	Coyote	Coyote
