# Supplementary material for: Combining phylogenetic and demographic inferences to assess the origin of the genetic diversity in an isolated wolf population
Source: PLoS One. 2017 May 10;12(5):e0176560. doi: 10.1371/journal.pone.0176560 (PMC5425034; doi:10.1371/journal.pone.0176560)
Supplement: S4 Table — (PDF) [file pone.0176560.s011.pdf]

S4 Table. DIYABC prior distributions for demographic parameters and mutation rates.

Parameter	Distribution	Minimum	Maximum	Mean	Shape
N1	UN	10	10.000		
N2	UN	10	10.000		
N3	UN	10	10.000		
N1b	UN	10	10.000		
N2b	UN	10	30.000		
N3b	UN	10	30.000		
N4	UN	10	30.000		
NA	UN	5	10.000		
t1	UN	500	10.000		
db	UN	10	10.000		
Mean μ	UN	1.0E-04	1.0E-03		
Gamma μ	GA	1.0E-05	1.0E-02	Mean_μ	2
Mean P	UN	1.0E-01	3.0E-01		
Gamma P	GA	1.0E-02	9.0E-01	Mean_P	2
Mean Sni	UN	1.0E-08	1.0E-05		
Gamma Sni	GA	1.0E-09	1.0E-04	Mean_Sni	2

Type of parameters: N= Effective population sizes; t=time (in generations); db=time of the bottleneck (in generations). Type of distributions: UN= uniform (between minimum and maximum values); GA=gamma-distributed (with indicated mean and gamma shape parameters).
