# Supplementary material for: Combining phylogenetic and demographic inferences to assess the origin of the genetic diversity in an isolated wolf population
Source: PLoS One. 2017 May 10;12(5):e0176560. doi: 10.1371/journal.pone.0176560 (PMC5425034; doi:10.1371/journal.pone.0176560)
Supplement: S5 Table — Detailed composition of the concatenated multifragment haplotypes. (PDF) [file pone.0176560.s012.pdf]

S5 Table. Haplotype composition. Detailed composition of the concatenated multifragment haplotypes**.**

MF	ATP6	COX3	NAD4	CR	MF	ATP6	COX3	NAD4	CR	MF	ATP6	COX3	NAD4	CR
**WH1**	A1	C1	N1	W1	**DH1**	A12	C1	N20	D1	**DH53**	A33	C3	N42	D60
**WH2**	A1	C1	N1	W2	**DH2**	A12	C1	N21	D1	**DH54**	A34	C18	N27	D60
**WH3**	A1	C1	N1	W3	**DH3**	A13	C1	N20	D1	**DH55**	A35	C18	N43	D61
**WH4**	A1	C2	N2	W4	**DH4**	A12	C1	N22	D1	**DH56**	A36	C3	N41	D61
**WH5**	A1	C1	N2	W4	**DH5**	A14	C1	N20	D1	**DH57**	A37	C18	N43	D61
**WH6**	A2	C1	N2	W5	**DH6**	A12	C1	N23	D1	**DH58**	A1	C1	N2	D62
**WH7**	A1	C1	N3	W7	**DH7**	A15	C1	N20	D1	**DH59**	A12	C1	N20	D62
**WH8**	A1	C1	N3	W8	**DH8**	A12	C1	N20	D2	**DH60**	A11	C19	N41	D63
**WH9**	A1	C1	N4	W9	**DH9**	A16	C11	N24	D5-6	**DH61**	A11	C3	N41	D63
**WH10**	A1	C1	N2	W10	**DH10**	A16	C11	N25	D5-6	**DH62**	A21	C3	N40	D64
**WH11**	A1	C1	N2	W11	**DH11**	A17	C11	N24	D5-6	**DH63**	A11	C3	N37	D64
**WH12**	A3	C3	N5	W13	**DH12**	A16	C11	N26	D5-6	**DH64**	A30	C3	N40	D64
**WH13**	A3	C3	N6	W13	**DH13**	A16	C11	N27	D5-6	**DH65**	A38	C20	N44	D65
**WH14**	A4	C4	N7	W14	**DH14**	A18	C11	N28	D8	**DH66**	A39	C21	N45	D66
**WH15**	A4	C4	N8	W14	**DH15**	A16	C11	N29	D8	**DH67**	A27	C11	N27	D67
**WH16**	A1	C1	N3	W14	**DH16**	A16	C11	N30	D8	**DH68**	A12	C1	N20	D68
**WH17**	A4	C4	N7	W15	**DH17**	A18	C11	N52	D8	**DH69**	A12	C1	N20	D69
**WH19**	A4	C4	N7	W16	**DH18**	A19	C12	N27	D9	**DH70**	A21	C11	N27	D70
**WH18**	A4	C4	N8	W16	**DH19**	A19	C11	N27	D9	**DH71**	A12	C1	N21	D71
**WH20**	A4	C4	N8	W17	**DH20**	A19	C11	N31	D9	**DH72**	A40	C13	N46	D72
**WH21**	A4	C4	N8	W18	**DH21**	A20	C13	N32	D10	**DH73**	A12	C1	N20	D73
**WH22**	A1	C5	N2	W19	**DH22**	A21	C11	N27	D11	**DH74**	A41	C22	N27	D74
**WH23**	A1	C5	N2	W20	**DH23**	A21	C11	N27	D13	**DH75**	A12	C1	N20	D75
**WH24**	A1	C5	N9	W20	**DH24**	A22	C11	N27	D13	**DH76**	A12	C1	N20	D76
**WH25**	A1	C5	N2	W21	**DH25**	A12	C1	N33	D13	**DH77**	A21	C11	N27	D77
**WH26**	A1	C1	N1	W22	**DH26**	A23	C11	N27	D13	**DH78**	A12	C1	N21	D78
**WH27**	A1	C6	N10	W23	**DH27**	A22	C11	N34	D13	**DH79**	A27	C11	N27	D79
**WH28**	A1	C1	N11	W24	**DH28**	A24	C11	N27	D13	**DH80**	A11	C3	N47	D80
**WH29**	A1	C1	N1	W24	**DH29**	A21	C14	N27	D14	**DH81**	A21	C11	N27	D81
**WH30**	A5	C3	N12	W25	**DH30**	A25	C14	N27	D14	**DH82**	A16	C23	N27	D82
**WH31**	A1	C1	N2	W26	**DH31**	A21	C11	N27	D14	**DH83**	A12	C1	N20	D83
**WH32**	A6	C1	N3	W27	**DH32**	A26	C15	N35	D51	**DH84**	A37	C18	N48	D84
**WH33**	A7	C7	N13	W28	**DH33**	A26	C15	N35	D52	**DH85**	A42	C13	N49	D85
**WH34**	A8	C1	N2	W29	**DH34**	A21	C11	N27	D53	**DH86**	A43	C15	N50	D86
**WH35**	A9	C1	N14	W30	**DH35**	A21	C16	N27	D53	**DH87**	A20	C13	N32	D87
**WH36**	A9	C1	N15	W30	**DH36**	A27	C11	N27	D53	**DH88**	A12	C1	N20	D88
**WH37**	A10	C1	N2	W31	**DH37**	A28	C11	N27	D53	**DH89**	A11	C3	N51	D89
**WH38**	A10	C1	N2	W32	**DH38**	A11	C3	N36	D54	**DH90**	A16	C11	N30	D90
**WH39**	A10	C1	N2	W33	**DH39**	A12	C1	N20	D55	**DH91**	A21	C11	N27	D91

**WH40**	A1	C1	N16	W34	**DH40**	A11	C3	N37	D56	**DH92**	A21	C11	N27	D92
**WH41**	A10	C1	N2	W35	**DH41**	A11	C3	N38	D56	**DH93**	A21	C11	N27	D93
**WH42**	A11	C8	N17	W36	**DH42**	A29	C3	N37	D56	**DH94**	A23	C11	N27	D94
**WH43**	A11	C8	N17	W37	**DH43**	A11	C3	N39	D56	**DH95**	A16	C11	N24	D95
**WH44**	A7	C7	N13	W38	**DH44**	A11	C3	N40	D56	**DH96**	A12	C1	N20	D96
**S14.5k**	A45	C4	N8	S14.5k	**DH45**	A30	C3	N37	D56	**DH97**	A12	C24	N20	D97
**R18k**	A46	C9	N18	R18k	**DH46**	A16	C17	N25	D57	**DH98**	A12	C1	N21	D98
**R22k**	A47	C3	N13	R22k	**DH47**	A16	C11	N25	D57	**DH99**	A11	C3	N37	D99
**B30k**	A48	C10	N19	B30k	**DH48**	A21	C11	N27	D58	**DH100**	A11	C3	N36	D100
					**DH49**	A31	C11	N27	D58	**DH101**	A21	C11	N53	D101
					**DH50**	A32	C15	N35	D59	**DH102**	A44	C13	N32	D102
					**DH51**	A26	C15	N35	D59	**Basenji**	A16	C11	N27	Basenji
					**DH52**	A11	C3	N41	D60	**Coyote**	A49	C25	N54	Coyote
