# Supplementary material for: Combining phylogenetic and demographic inferences to assess the origin of the genetic diversity in an isolated wolf population
Source: PLoS One. 2017 May 10;12(5):e0176560. doi: 10.1371/journal.pone.0176560 (PMC5425034; doi:10.1371/journal.pone.0176560)
Supplement: S6 Table — Country acronyms: IB = Iberian Peninsula; IT = Italy; HR = Croatia; SL = Slovenia; GR = Greece; BG = Bulgaria; PL = Poland; ES = Estonia; LA = Latvia; FI = Finland; UK = Ukraine; SW = Sweden; IR = Iran; OM = Oman; SA = Saudi Arabia; IS = Israel; IN = India; CH = China; AK = Alaska; CA = Canada; US = USA; MX = Mexico. (PDF) [file pone.0176560.s013.pdf]

S6 Table. Distribution of the concatenated multifragment mtDNA haplotypes in wolves sampled all over the world. Country acronyms: IB = Iberian Peninsula; IT= Italy; HR = Croatia; SL = Slovenia; GR = Greece; BG = Bulgaria; PL = Poland; ES = Estonia; LA = Latvia; FI = Finland; UK = Ukraine; SW = Sweden; IR = Iran; OM = Oman; SA = Saudi Arabia; IS = Israel; IN = India; CH = China; AK = Alaska; CA = Canada; US = USA; MX = Mexico.

	IB	IT	HR	SL	GR	BG	PL	ES	LA	FI	UK	SW	IR	OM	SA	IS	IN	CH	AK	CA	US	MX	TOT
WH1	-	-	-	-	-	1	-	-	-	-	-	-	-	-	-	-	-	-	-	-	-	-	1
WH2	-	-	-	-	-	1	-	-	-	-	-	-	-	-	-	-	-	-	-	-	-	-	1
WH3	-	-	-	16	-	-	-	-	-	-	-	-	-	-	-	-	-	-	-	-	-	-	16
WH4	-	-	-	-	4	-	-	-	-	-	-	-	-	-	-	-	-	-	-	-	-	-	4
WH5	-	-	-	-	-	-	-	-	-	-	1	-	-	-	-	-	-	-	-	-	-	-	1
WH6	-	-	-	-	4	5	-	-	-	-	-	-	-	-	-	-	-	-	-	-	-	-	9
WH7	-	-	-	-	-	-	7	7	9	3	-	-	-	-	-	-	-	-	-	-	-	-	26
WH8	-	-	-	-	-	-	-	-	-	3	-	-	-	-	-	-	-	-	-	-	-	-	3
WH9	-	-	-	-	-	5	2	-	-	-	-	-	-	-	-	-	-	-	-	-	-	-	7
WH10	-	-	1	-	-	-	-	-	-	-	-	-	-	-	-	-	-	-	-	-	-	-	1
WH11	-	-	-	-	-	-	-	-	-	-	-	-	-	-	-	1	-	-	-	-	-	-	1
WH12	-	-	-	-	-	-	-	-	-	5	-	-	-	-	-	-	-	-	-	-	-	-	5
WH13	-	-	-	-	-	-	-	-	-	2	-	-	-	-	-	-	-	-	-	-	-	-	2
WH14	-	34	-	-	-	-	-	-	-	-	-	-	-	-	-	-	-	-	-	-	-	-	34
WH15	-	-	-	-	1	-	-	-	-	-	-	-	-	-	-	-	-	-	-	-	-	-	1
WH16	-	-	-	-	2	-	-	-	-	-	-	-	-	-	-	-	-	-	-	-	-	-	2
WH17	-	-	-	-	2	-	-	-	-	-	-	-	-	-	-	-	-	-	-	-	-	-	2
WH18	-	-	-	-	2	5	-	-	-	-	-	-	-	-	-	-	-	-	-	-	-	-	7
WH19	-	5	-	-	-	-	-	-	-	-	-	-	-	-	-	-	-	-	-	-	-	-	5
WH20	-	-	-	4	-	-	-	-	-	-	-	-	-	-	-	-	-	-	-	-	-	-	4
WH21	-	-	-	-	-	-	1	-	-	-	-	-	-	-	-	-	-	-	-	-	-	-	1
WH22	7	-	-	-	-	-	-	-	-	-	-	-	-	-	-	-	-	-	-	-	-	-	7
WH23	4	-	-	-	-	-	-	-	-	-	-	-	-	-	-	-	-	-	-	-	-	-	4
WH24	4	-	-	-	-	-	-	-	-	-	-	-	-	-	-	-	-	-	-	-	-	-	4
WH25	4	-	-	-	-	-	-	-	-	-	-	-	-	-	-	-	-	-	-	-	-	-	4
WH26	-	-	-	-	-	-	1	-	-	-	-	-	-	-	-	-	-	-	-	-	-	-	1
WH27	-	-	-	-	-	-	5	1	1	-	-	-	-	-	-	-	-	-	-	-	-	-	7
WH28	-	-	-	-	-	-	-	1	-	-	-	-	-	-	-	-	-	-	-	-	-	-	1
WH29	1	-	-	-	-	-	-	-	-	-	-	-	-	-	-	-	-	-	-	-	-	-	1
WH30	-	-	-	-	-	-	-	-	-	-	-	-	-	-	1	-	-	-	-	-	-	-	1
WH31	-	-	-	-	-	-	-	-	-	-	-	-	-	-	-	-	-	1	-	-	-	-	1
WH32	-	-	-	-	-	-	-	-	-	-	-	1	-	-	-	-	-	-	-	-	-	-	1
WH33	-	-	-	-	-	-	-	-	-	-	-	-	1	-	-	-	-	-	-	-	-	-	1
WH34	-	-	-	-	-	-	-	-	-	-	-	-	-	-	-	-	1	-	-	-	-	-	1
WH35	-	-	-	-	-	-	-	-	-	-	-	-	-	-	-	-	-	-	-	1	-	-	1
WH36	-	-	-	-	-	-	-	-	-	-	-	-	-	-	-	-	-	-	-	1	-	-	1
WH37	-	-	-	-	-	-	-	-	-	-	-	-	-	-	-	-	-	-	-	1	-	-	1
WH38	-	-	-	-	-	-	-	-	-	-	-	-	-	-	-	-	-	-	1	-	-	-	1
WH39	-	-	-	-	-	-	-	-	-	-	-	-	-	-	-	-	-	-	1	-	-	-	1
WH40	-	-	-	-	-	-	-	-	-	-	-	-	-	-	-	-	-	-	1	-	-	-	1
WH41	-	-	-	-	-	-	-	-	-	-	-	-	-	-	-	-	-	-	-	-	1	-	1
WH42	-	-	-	-	-	-	-	-	-	-	-	-	-	-	-	-	-	-	-	-	-	1	1
WH43	-	-	-	-	-	-	-	-	-	-	-	-	-	-	-	-	-	-	-	-	-	1	1
WH44	-	-	-	-	-	-	-	-	-	-	-	-	-	1	-	-	-	-	-	-	-	-	1
Total	20	39	1	20	15	17	16	9	10	13	1	1	1	1	1	1	1	1	3	3	1	2	177
