# Supplementary material for: Combining phylogenetic and demographic inferences to assess the origin of the genetic diversity in an isolated wolf population
Source: PLoS One. 2017 May 10;12(5):e0176560. doi: 10.1371/journal.pone.0176560 (PMC5425034; doi:10.1371/journal.pone.0176560)
Supplement: S7 Table — (PDF) [file pone.0176560.s014.pdf]

S7 Table. Model checking results for the best fitting scenario (SC2) based on 1,000 simulated datasets.

Summary statistics	Observed value	p-value ($S_{simul.} < S_{obs.}$) sc2
NAL_1_1	4.359	0.108
NAL_1_2	4.641	0.162
NAL_1_3	5.641	0.215
HET_1_1	0.515	0.115
HET_1_2	0.633	0.235
HET_1_3	0.687	0.332
VAR_1_1	0.723	**0.042***
VAR_1_2	0.870	0.051
VAR_1_3	0.892	0.052
N2P_1_1&2	6.333	0.054
N2P_1_1&3	6.949	0.072
N2P_1_2&3	6.692	0.075
H2P_1_1&2	0.650	0.117
H2P_1_1&3	0.681	0.139
H2P_1_2&3	0.731	0.251
FST_1_1&2	0.273	0.808
FST_1_1&3	0.229	0.737
FST_1_2&3	0.165	0.573
DAS_1_1&2	0.216	0.864
DAS_1_1&3	0.221	0.872
DAS_1_2&3	0.205	0.844

*; all p-values were non-significant after applying Bonferroni correction for multiple testing. Abbreviations for the summary statistics are as follows: NAL= mean number of alleles across loci; HET= mean gene diversity across loci; VAR= mean allele size variance across loci; N2P= mean number of alleles across loci (two samples); H2P= mean gene diversity across loci (two samples); FST= FST between two samples; DAS= shared allele distance between two samples.
