# Supplementary material for: Combining phylogenetic and demographic inferences to assess the origin of the genetic diversity in an isolated wolf population
Source: PLoS One. 2017 May 10;12(5):e0176560. doi: 10.1371/journal.pone.0176560 (PMC5425034; doi:10.1371/journal.pone.0176560)
Supplement: S1 Appendix — (PDF) [file pone.0176560.s015.pdf]

A) Description of laboratory methods with details on primers and PCR profiles for all the genotyped markers

The 39 canine autosomal STRs includes: 1) 12 STRs used in a 10-years long non-invasive wolf monitoring project in Italy [1]; 2) 12 STRs used in a hybridization study by Godinho et al. [2]; and 3) 15 STRs from the Finnzymes Canine multiplex kit (Finnzymes, Thermo Scientific Canine Genotypes™); one of them, the *Amelogenin* marker, was used to sex the individuals (the other 4 of the 19 STRs included in this kit were not used because they showed confusing electropherograms).

The 24 autosomal STR loci were amplified in other 4 multiplexed primer mixes (M1, M2, M3, M4) using the Qiagen Multiplex PCR Kit (Qiagen Inc, Hilden, Germany), an ABI GeneAmp® PCR System 9700, and the following thermal profile: 94°C/15 min, 94°C/30 sec, 57°C/90 sec, 72°C/60 sec (30 cycles), followed by a final extension step at 72°C for 5 min. Amplifications were carried out in 10 µl total volume, including 2 µl of DNA solution from saliva samples, or 1 µl of DNA solution from muscle and blood samples, 5 µl Qiagen Multiplex PCR mix, 1 µl Qiagen Q solution, 0.4 µM deoxynucleotide triphosphates (dNTP), from 0.1 µl to 0.4 µl of 10 µM primer mix (forward and reverse) and RNase-free water up to the final volume.

The microsatellites in the Finnzymes Canine multiplex kit (Finnzymes, Thermo Scientific Canine Genotypes™) were amplified in a single multiplex PCR reaction (MF) using an Applied Biosystems Thermal Cycler (ABI GeneAmp® PCR System 9700) with the following thermal profile: 98°C/3 min, 98°C/15 sec, 60°C/90 sec, 72°C/30 sec (30-40 cycles), followed by a final extension step at 72°C for 5 min. The amplifications were carried out in a 20 µl total PCR volume, including 2 µl of DNA solution from saliva samples, or 1 µl of DNA solution from muscle and blood samples, corresponding to *c*. 20 – 40 ng of DNA, 10 µl of Finnzymes Canine Genotypes™ Panel 1.1 Master Mix (which included an optimized buffer containing MgCl2, deoxynucleoside triphosphates (dNTP) and Phusion™ Hot Start DNA Polymerase with an activity of 0.05 U/µl), and

10 μl of Finnzymes Canine Genotypes™ Panel 1.1 Primer Mix (including forward and reverse primers for the 19 markers).

B) Details on MrBayes and Beast models

The best fit evolutionary models for MrBayes scheme are: HKY for COX III; HKY + I for ATPase 6 and ND4; HKY + I + G for CR and multi-fragment alignments. The best-fit evolutionary models for BEAST v2.1.3. single-fragment schemes are:

- ATPase 6: HKY + G for 1st and 2nd bases (G category= 4, G shape = 0.21; Kappa= 42.96); TrN for 3rd bases (Kappa1(purines)= 41.13; Kappa2(pyrimidines)= 9.267);
- COX III: HKY + I for 1st and 2nd bases (I= 0.216; Kappa= 16.175); HKY for 3rd bases (Kappa= 54.147);
- ND4: HKY + I for 1st and 2nd bases (I= 0.0744; Kappa= 10.505); HKY for 3rd bases (Kappa= 37.0);
- CR: HKY for all the bases (I= 0.5446; G category= 4, G shape = 0.73; Kappa= 70.28);

The best-fit evolutionary models for BEAST multi-fragment (MF) schemes are: TrN + G for ATPase 6, COX III, and ND4 3rd bases (G category= 4, G shape = 1,7038; Kappa1(purines)= 39,61; Kappa2(pyrimidines)= 13,507); HKY + I for ATPase 6, COX III, and ND4 1st and 2nd bases (I = 0,11; Kappa=20,11); HKY + I + G for CR (I= 0,5446; G category= 4, G shape = 0,73; Kappa= 70,28). Kappa, I (proportion of invariants), and G (gamma) parameters were calculated using MEGA6 (Tamura et al., 2013).]

References

1. Caniglia R, Fabbri E, Cubaynes S, Gimenez O, Lebreton J-D, Randi E. An improved procedure to estimate wolf abundance using non-invasive genetic sampling and capture-recapture mixture models. Conserv Genet. 2012;13: 53–64. doi:10.1007/s10592-011-0266-1

2. Godinho R, Llaneza L, Blanco JC, Lopes S, Álvares F, García EJ, et al. Genetic evidence for multiple events of hybridization between wolves and domestic dogs in the Iberian Peninsula. Mol Ecol. 2011;20: 5154–5166. doi:10.1111/j.1365-294X.2011.05345.x
